# Constraining regulatory domain dynamics of the Src kinase Fgr increases ATP-site inhibitor sensitivity and impairs bone marrow engraftment

**DOI:** 10.1016/j.celrep.2026.117551

**Published:** 2026-06-16

**Authors:** Giancarlo Gonzalez-Areizaga, Sherry T. Shu, John J. Alvarado, Haibin Shi, Li Chen, Thomas E. Smithgall

**Affiliations:** 1 Department of Microbiology and Molecular Genetics, University of Pittsburgh School of Medicine, Pittsburgh, PA 15219, USA; 2 Lead contact

## Abstract

Acute myeloid leukemia is often associated with constitutive activation of the Src-family kinases, Hck, Lyn, and Fgr. Their modular SH3 and SH2 domains regulate kinase activity and signal transduction. Here, we show that regulatory domain dynamics critically influence both inhibitor sensitivity and leukemogenic signaling. We modified the Fgr SH2-kinase linker to enhance intramolecular SH3 engagement, shifting the conformational ensemble to the closed state. This shift increased the K_m_ for ATP and enhanced the potency of ATP-site inhibitors *in vitro*. Human myeloid cells expressing these constrained Fgr variants exhibited heightened sensitivity to ATP-site inhibitors in terms of growth arrest. These cells also demonstrated impaired bone marrow engraftment *in vivo*, suggesting a key role for Fgr dynamics and SH3-dependent signaling in leukemia cell survival within this niche. Small molecules that similarly restrict Src-family kinase regulatory domain dynamics may provide a new therapeutic approach to AML and other cancers linked to these kinases.

## In brief

Acute myeloid leukemia often involves overactive signaling proteins including Src-family kinases. Gonzalez-Areizaga et al. show that restricting the dynamics of the AML-associated kinase Fgr increases its inhibitor sensitivity. AML cells expressing restricted Fgr variants show reduced survival in bone marrow, suggesting a new strategy for treating this aggressive blood cancer.

## Graphical abstract

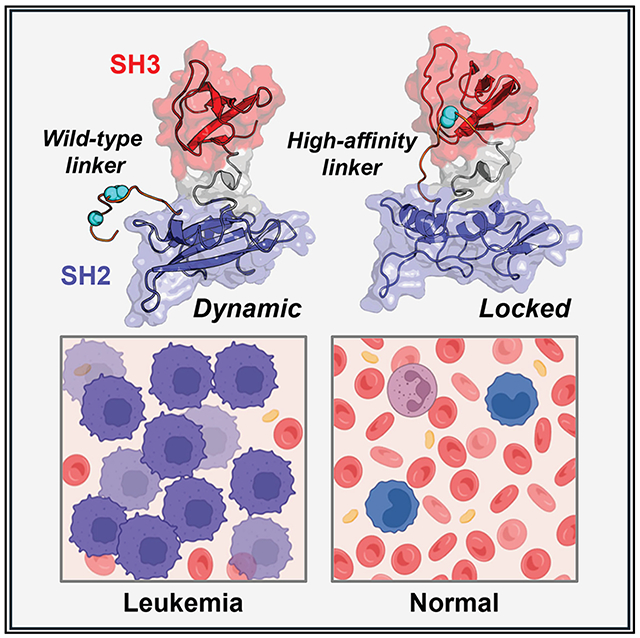

## INTRODUCTION

Of the various genetic factors related to the development of acute myeloid leukemia (AML), upregulation of tyrosine kinase signaling pathways remains as one of the most notable features linked to this devastating disease. A predominant example, observed in about one-third of all AML cases, involves activating mutations in the Flt3 receptor tyrosine kinase, leading to the uncontrolled proliferation and survival of leukemic cells.^1^ While multiple Flt3 kinase inhibitors have been developed for the clinic, none have produced durable responses primarily because of acquired resistance mutations within the Flt3 kinase domain.^2,3^ In addition to FLT3, AML is often associated with overexpression of non-receptor tyrosine kinases, including the three Src-family kinases (SFKs) Hck, Lyn, and Fgr. Like Flt3, high expression levels of these SFKs correlate with worse AML patient outcomes,^4^ making them compelling alternative targets for anti-AML drug development.

All Src-family members share a common domain organization consisting of a myristoylated N-terminal unique region followed by the conserved Src-homology (SH) regulatory domains, SH3 and SH2. These domains join the bilobed kinase domain via a linker that forms a polyproline type II (PPII) helix. A regulatory tail extends from the C terminus of the kinase domain with a conserved tyrosine phosphorylation site essential for negative regulation. This site is phosphorylated primarily by a separate regulatory kinase known as Csk.^5^ The intrinsically disordered N-terminal domain is essential for membrane localization, and is the only region with high sequence variability across the Src kinase family.^6^ Crystal structures of near-full-length Src,^7,8^ Hck,^9–11^ and Fgr^12^ show that the SH3 domain engages the PPII helix formed by the SH2-kinase linker, while the tyrosine-phosphorylated C-terminal tail engages the SH2 domain. Together, these intramolecular interactions hold the kinase in a closed conformation. Moreover, mutations or peptide ligands that disrupt SH3- and SH2-mediated interactions induce “opening” of the kinase conformation, often leading to increased enzymatic activity.^13^ These findings have led to the idea that all SFKs are negatively regulated by intramolecular interactions involving their SH3 and SH2 domains, and that phosphorylation of the negative regulatory tail is required for downregulation of kinase activity in cells. One exception to this general principle is Fgr, in which regulatory domain displacement does not affect kinase activity, even though the kinase is phosphorylated on the tail and can adopt the closed conformation.^14^

Previous studies have identified ATP-site inhibitors of myeloid SFKs with significant anti-AML efficacy. These inhibitors include the pyrrolopyrimidine A-419259 and the N-phenylbenzamide TL02-59; the latter compound inhibited Fgr *in vitro* with enhanced potency compared to other SFKs and reversed bone marrow engraftment of the human AML cell line MV4-11 in a mouse model of AML.^4,15,16^ Recently, we reported X-ray crystal structures of near-full-length Fgr (SH3-SH2-kinase tail) with each of these inhibitors bound to the active site.^12^ With A-419259, the regulatory SH3 and SH2 domains packed against the back of the kinase domain, resulting in a closed conformation observed in previous structures of Hck with this inhibitor.^11^ Surprisingly, TL02-59 induced allosteric displacement of the SH3 and SH2 domains from their regulatory positions, resulting in an open conformation that was confirmed in solution by hydrogen-deuterium exchange mass spectrometry.^12^ Thus, we hypothesized that the allosteric displacement of the SH3 and SH2 domains from the Fgr kinase domain may be an important feature of TL02-59 potency and other inhibitors that bind via conformationally dependent “type II” mechanisms.^17^

In the present study, we investigated the role of conformational flexibility on Fgr signaling and sensitivity to ATP-site inhibitors both *in vitro* and *in vivo*. Guided by previous X-ray crystal structures, we engineered forms of Fgr that enhanced the internal interaction of the SH3 domain with the SH2-kinase linker. Structural, biophysical, and biochemical analyses confirmed that these linker mutations, termed high affinity linkers (HALs), increased SH3-linker interaction both *in vitro* and *in vivo*. Restriction of Fgr regulatory domain dynamics in this manner increased the K_m_ for ATP and enhanced the inhibitory potency of both TL02-59 and A-419259 with recombinant kinases *in vitro*, suggesting that both ATP-site inhibitors prefer the closed Fgr conformation. This result is surprising given the distinct binding mode of each inhibitor in the ATP site of the kinase domain (A-419259, DFG-in/C-helix out; TL02-59, DFG-out/C-helix in; “type II”) and our previous finding that TL02-59 causes allosteric disturbance of regulatory domain packing.^12^ We also transformed the human AML cell line TF-1 to cytokine independence with active forms of Fgr bearing wild-type (WT) or HALs. Here again, stabilization of the closed conformation enhanced ATP-site inhibitor potency in terms of growth suppression. TF-1 cell populations expressing WT vs. HAL versions of Fgr showed similar levels of tumor burden following injection of immunocompromised mice by *in vivo* image analysis. However, cells expressing a HAL variant of Fgr showed significantly decreased bone marrow engraftment compared to wild type, suggesting the possibility that restriction of SH3 domain access may interfere with signaling necessary for cell survival in this compartment. These observations suggest that small molecule SH3 antagonists for Fgr and other AML-associated SFKs may provide a unique therapeutic opportunity.

## RESULTS

### Structure-guided design of Fgr variants with restricted regulatory domain dynamics

**Figure 1.**
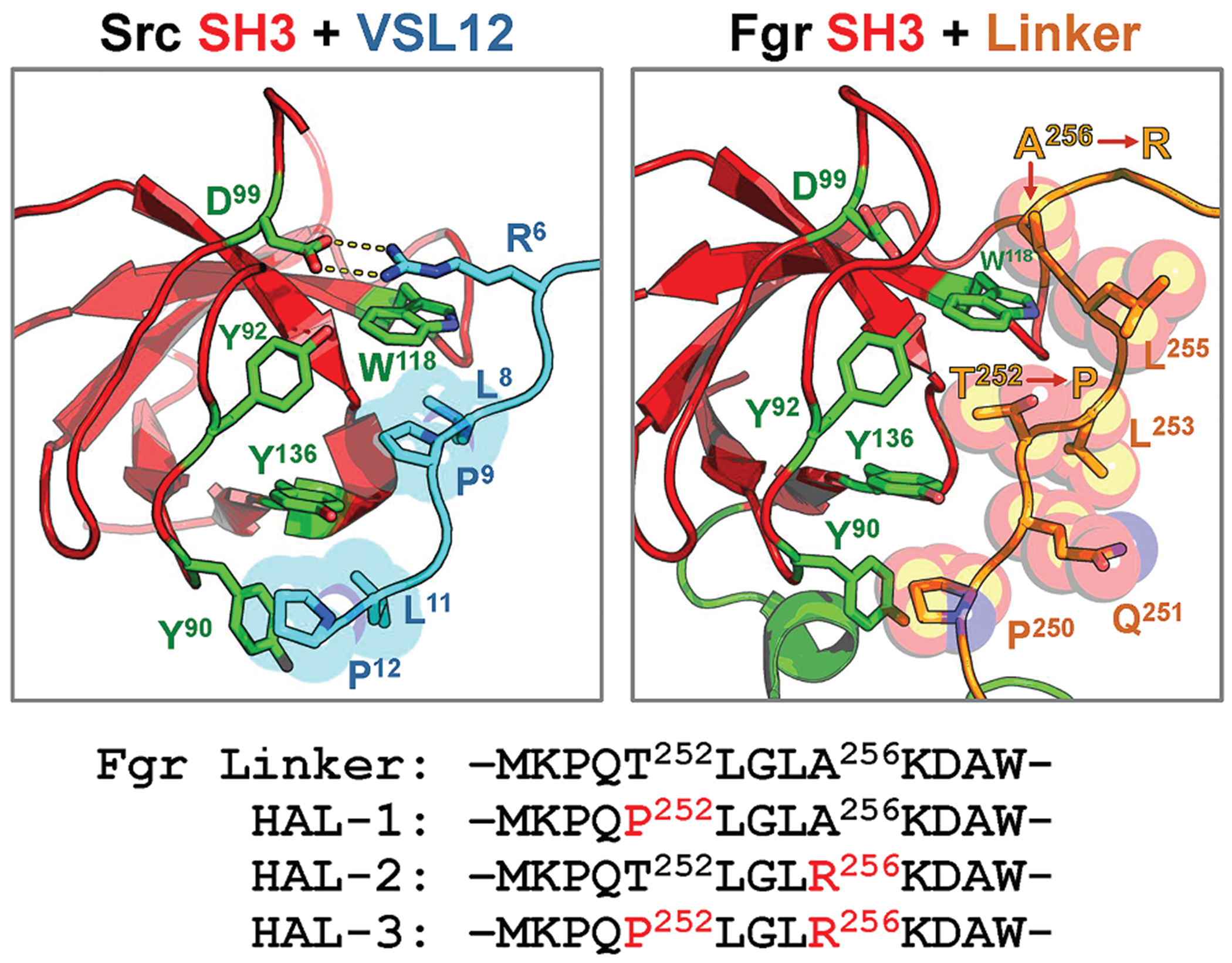
Structure-based design of HAL variants to enhance Fgr SH3:linker interaction (*Top*) Comparison of the crystal structures of the Src SH3 domain bound to the optimized peptide ligand, VSL12 (*left*), with the Fgr SH3-linker interface (*right*). Specific substitutions were made to the linker region of Fgr to mimic the interactions between VSL12 and the SH3 domain. Replacement of T252 with proline favors hydrophobic interaction with the SH3 surface groove formed by the side chains of Y92 and Y136, while substitution of A256 with arginine is predicted to form a salt bridge with SH3 residue D99. T252P was designated as Fgr HAL-1, A256R as Fgr HAL-2 and double mutant T252P/A256R as HAL-3. Linker sequences are shown below the models with substitutions in red. Models were produced using the crystal structure of the Src SH3 domain in complex with VSL12 (PDB: 4RTZ) and the Fgr SH3:linker interface from the cocrystal structure with A-419259 (PDB: 7UY0).

To better understand the effects of Fgr regulatory domain dynamics on signaling and ATP-site inhibitor action, we designed Fgr variants expected to stabilize a closed overall kinase conformation. To do this, we modified the SH2-kinase linker region to enhance its intramolecular interaction with the SH3 domain (HAL variants). Our design was based on a comparison of a previous crystal structure of the Src SH3 domain bound to an optimized SH3-binding peptide (VSL12; protein data bank [PDB]: 4RTZ) with the native SH3:linker interface of Fgr in the closed conformation (PDB: 7UY0; Figure 1). This comparison suggested that substitution of linker Thr252 with proline (T252P) would favor hydrophobic interaction with the SH3 surface groove between Tyr92 and Tyr136, while replacement of linker Ala256 with arginine (A256R) would form a stabilizing salt bridge with SH3 residue Asp99. Three versions were created with either one or both substitutions. We designated T252P as Fgr HAL-1, A256R as Fgr HAL-2 and the double mutant T252P/A256R as HAL-3. All residue numbering is based on the crystal structure of Fgr in the closed conformation (PDB: 7UY0).

### Crystal structures of wild-type vs. HAL SH3-SH2-linker proteins

**Figure 2.**
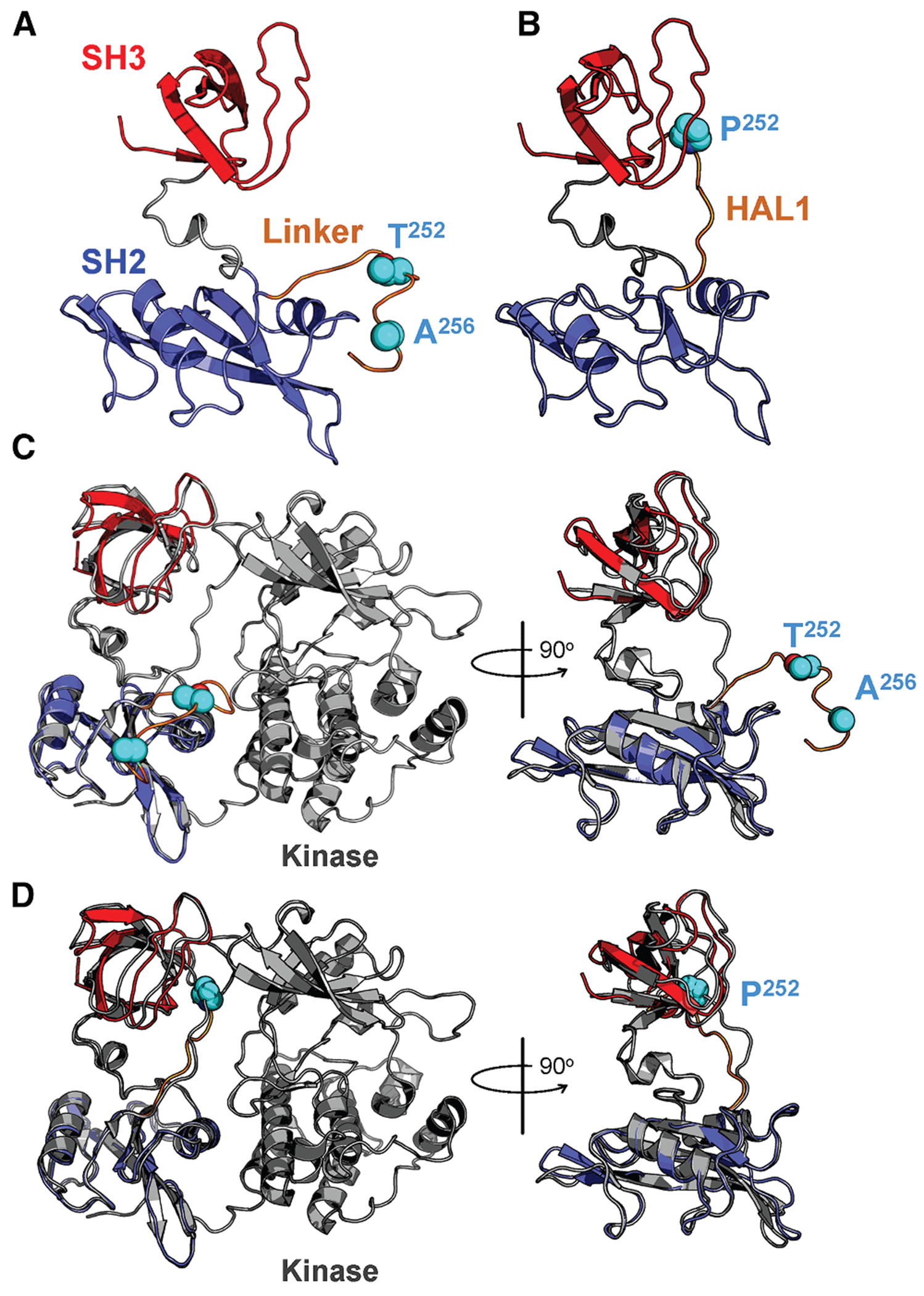
X-ray crystal structures of WT and HAL1 SH3-SH2-linker (32L) proteins (A) Structure of WT 32L protein showing the SH3 domain (red), SH2 domain (blue), and linker (orange). Linker residues substituted in the HAL variants are highlighted in cyan (T252 and A256). (B) Structure of Fgr 32-HAL1 in which T252 is replaced with proline. Unlike the WT structure, the HAL1 linker contacts the SH3 domain. (C) Alignment of WT 32L and near-full-length Fgr (PDB: 7UY0; rendered in gray) structures. Without the kinase domain, the linker does not contact the SH3 domain and extends outward (shown more clearly in the rotated model at right). (D) Alignment of 32-HAL1 with near-full-length Fgr. In this case the modified linker adopts a conformation compatible with the closed conformation of the overall kinase. The root mean square deviation between 32-HAL1 and 32L in 7UY0 is 0.8 Å over 140 α-carbon atoms. In the rotated alignments (C and D, right), the kinase domains are not shown for clarity.

To explore the impact of the HAL substitutions on Fgr SH3:linker interaction, we first expressed and purified recombinant SH3-SH2-linker (32L) proteins with WT or HALs for X-ray crystallography. WT Fgr 32L as well as all three HAL variants readily formed diffracting crystals, and X-ray structures were determined (Table S1). In each of the four structures, the SH3 domain, the SH3-SH2 connector, and the SH2 domain are clearly resolved. In the absence of the kinase domain, however, the WT linker extends outward and does not contact the SH3 domain (Figure 2A). However, replacement of Thr252 with proline in the HAL-1 variant reordered the linker, resulting in SH3 domain engagement (Figure 2B). We then aligned the 32L structures with the structure of the closed conformation of near-full-length Fgr, which also includes the kinase domain and the negative regulatory tail (PDB: 7UY0^12^). Alignment with WT 32L confirms that in the absence of the kinase domain, the linker is unstructured and adopts a conformation incompatible with SH3 engagement (Figure 2C). On the other hand, alignment with 32-HAL-1 reveals a linker conformation close to that observed in the near-full-length structure, demonstrating that this single point mutation is sufficient to structure the linker for SH3 engagement, despite the absence of the kinase domain. In the crystal structures of the 32-HAL-2 and 32-HAL-3 proteins (Figure S1A), the SH3 and SH2 domains are oriented in the same conformation as observed in the closed conformation of near-full-length Fgr following alignment (Figure S1B). However, in the HAL-2 and HAL-3 structures, both of which substitute linker Ala256 with arginine, a crystal contact is present between Asp99 in the SH3 domain RT-loop and Arg160 in the SH2 domain of a symmetry related molecule. This crystal contact precluded intramolecular interaction of the SH3 domain with the HAL. However, N-terminal amino acids are observed for both linkers which align very closely with the analogous residues in the linker of near full-length Fgr (Figure S1C), suggesting that the overall conformation of full-length HAL-2 and HAL-3 is the same as WT. Biophysical experiments described in the next section provide clear evidence for enhanced SH3 interaction with all three linker variants.

### HAL mutations occlude the Fgr SH3 domain binding surface

**Figure 3.**
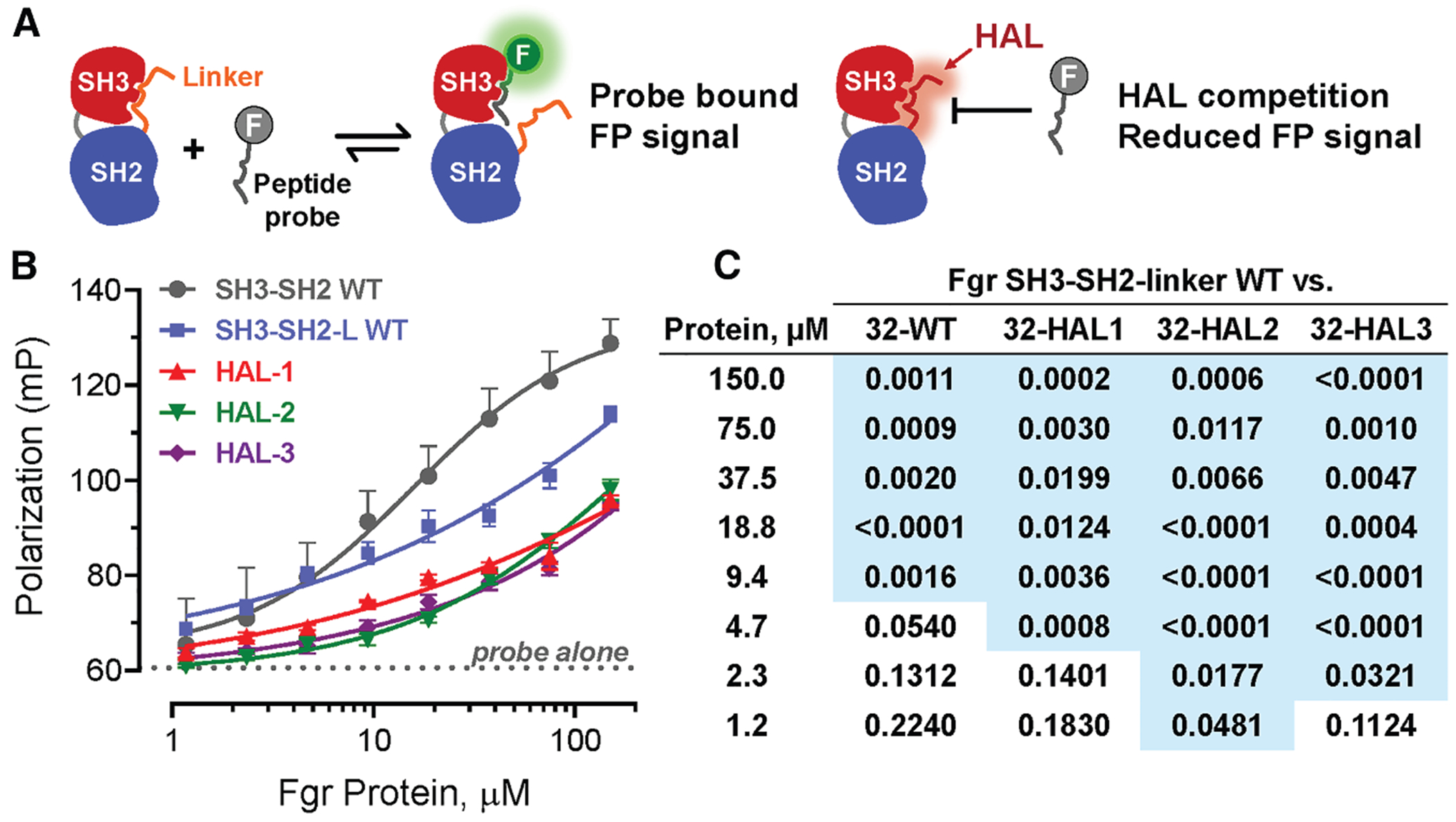
Introduction of HAL mutations occludes the Fgr SH3 domain binding surface in a fluorescence polarization (FP) assay (A) Schematic of the FP assay used to assess Fgr SH3-SH2-HAL protein binding to the fluorescent SH3-binding peptide, VSL12. (B) FP binding curves of Fgr SH3-SH2, SH3-SH2-linker (SH3-SH2-L), and the three Fgr SH3-SH2-HAL proteins with a fixed concentration of the fluorescent probe peptide (1.25 μM). Each point represents the mean of four technical replicates ±SEM. (C) FP values for the SH3-SH2-linker WT protein were compared with SH3-SH2 (32-WT) and each of the SH3-SH2-HAL proteins at each protein concentration by one-way ANOVA. The resulting *p* values are shown in the table; comparisons reaching statistical significance defined as *p* < 0.05 are highlighted in light blue.

We next evaluated whether HAL substitutions enhance SH3 engagement in *cis* with the same Fgr 32-HAL proteins used for crystallography. We have shown previously that the SH3-binding peptide VSL12, described above, outcompetes intramolecular SH3-linker interaction in recombinant downregulated Src and Hck, resulting in SH3 domain displacement and activation of the kinase domain.^14,18^ Based on this principle, we reasoned that introduction of HAL mutations in Fgr would enhance linker interaction with the SH3 domain, resulting in reduced interaction with VSL12 when compared to the WT linker. To test this hypothesis, we performed fluorescence polarization experiments using fluorescently tagged VSL12 peptide as the reporter and monitored changes in the polarization signal as a function of each Fgr protein concentration (Figure 3A). In this assay, higher polarization signals correspond to an increase in the amount of VSL12 peptide bound to the target protein due to decreased tumbling velocity. All three HAL variants displayed significantly lower polarization signals when compared to the WT Fgr SH3-SH2-linker protein, which in turn showed lower polarization when compared to an Fgr SH3-SH2 protein without the linker (Figure 3B). Statistical comparisons were performed by one-way ANOVA and the resulting *p* values for each comparison are summarized in Figure 3C.

So far, we have demonstrated that HAL substitutions in Fgr SH3-SH2-linker proteins are consistent with domain and residue conformations for enhanced SH3-linker engagement that occlude the SH3 polyproline binding surface. However, these constructs exclude the kinase domain, which also impacts linker conformation and may influence the modified linker structure in a way not reflected in the shorter regulatory domain proteins. To address this point, we expressed and purified recombinant near full-length Fgr with both WT and HAL-3 modified linkers. This form of Fgr consists of the SH3, SH2, and kinase domains as well as the tyrosine-phosphorylated C-terminal tail, with a His-6 purification tag replacing the N-terminal unique region. We then tested the interaction kinetics of both WT and HAL-3 Fgr against the VSL12 peptide using surface plasmon resonance (SPR). For this experiment, we immobilized three Fgr proteins on the SPR chip: near full-length WT Fgr, near full-length Fgr HAL-3, and Fgr SH3-SH2. The VSL12 peptide was then injected at varying concentrations until steady state was reached, followed by a dissociation phase. Representative sensorgrams and the resulting kinetic constants for two independent experiments are shown in Figures S2A–S2C. The association rate constants (kon) for VSL12 were higher for WT Fgr vs. HAL-3 by a factor of 2.5 to 3-fold, consistent with the antagonistic effect of the HAL on SH3 accessibility to VLS12. The slower kinetics of VSL12 binding to HAL-3 vs. WT are evident in the initial phase of the interaction as shown in Figure S2D. Interestingly, the dissociation rate constants (k_off_) were slightly lower for HAL-3, suggesting that the HAL-3 complex with VSL12 is more stable once formed. The average of the two resulting K_D_ values, calculated from the ratio of k_off_ to k_on_, reflect an overall lower affinity of VSL12 for HAL-3 (K_D_ = 84.9 ± 18.0 μM) vs. WT (K_D_ = 54.8 ± 17.2 μM) Fgr. The Fgr SH3-SH2 protein showed the strongest binding affinity for VSL12 (K_D_ = 8.3 ± 1.6 μM). In this protein, the SH3 domain is fully exposed, and the peptide dissociates more slowly due to the absence of internal competition from the SH2-kinase linker.

### Introduction of HAL-3 increases the K_m_ for ATP and enhances ATP-site inhibitor action *in vitro*

Structural and biophysical data described above demonstrate that the HAL modifications shift the conformational equilibrium of Fgr toward a closed or locked conformation. We next investigated whether these changes impacted basal kinase activity. To achieve this, we performed *in vitro* kinase activity assays with near full-length WT Fgr and HAL-3 over a range of ATP concentrations with a constant amount of kinase protein and substrate peptide. These experiments used a kinetic kinase assay with an ATP regenerating system that reports the production of ADP from ATP as a function of peptide tyrosine phosphorylation.^18,19^ Interestingly, restriction of regulatory domain dynamics in the HAL-3 kinase variant resulted in a decreased basal kinase activity when compared to WT, with a 3-fold increase in the apparent Michaelis-Menten constant for ATP (K_m_ WT, 9.61 ± 4.04 μM; K_m_ HAL-3, 29.1 ± 2.99 μM; Figure S3A). This result was somewhat surprising in light of our previous findings that, unlike other SFKs, WT Fgr kinase activity is decoupled from regulatory domain control.^14^

We next explored the consequences of allosteric restriction on ATP-site inhibitor sensitivity using the same *in vitro* kinase assay. For these experiments, ATP was added in excess relative to the K_m_ for each kinase, and the ATP-site inhibitors, A-419259 and TL02-59, were assayed over a range of concentrations. Under these conditions, Fgr HAL-3 was more sensitive to both A-419259 (Figure S3B) and TL02-59 (Figure S3C) with a 2-fold decrease in the IC_50_ values for both inhibitors when compared to WT Fgr. The maximum reaction velocity was also decreased by both inhibitors in Fgr HAL-3, suggesting that a mixed inhibitory mechanism results from the presence of the modified linker. The enhanced inhibitor sensitivity of HAL-3 may relate in part to the ATP K_m_ effect as well as a possible impact of enhanced SH3-linker interaction on the inhibitor binding site.

### Fgr HAL proteins maintain enhanced SH3-linker interaction in AML cells

**Figure 4.**
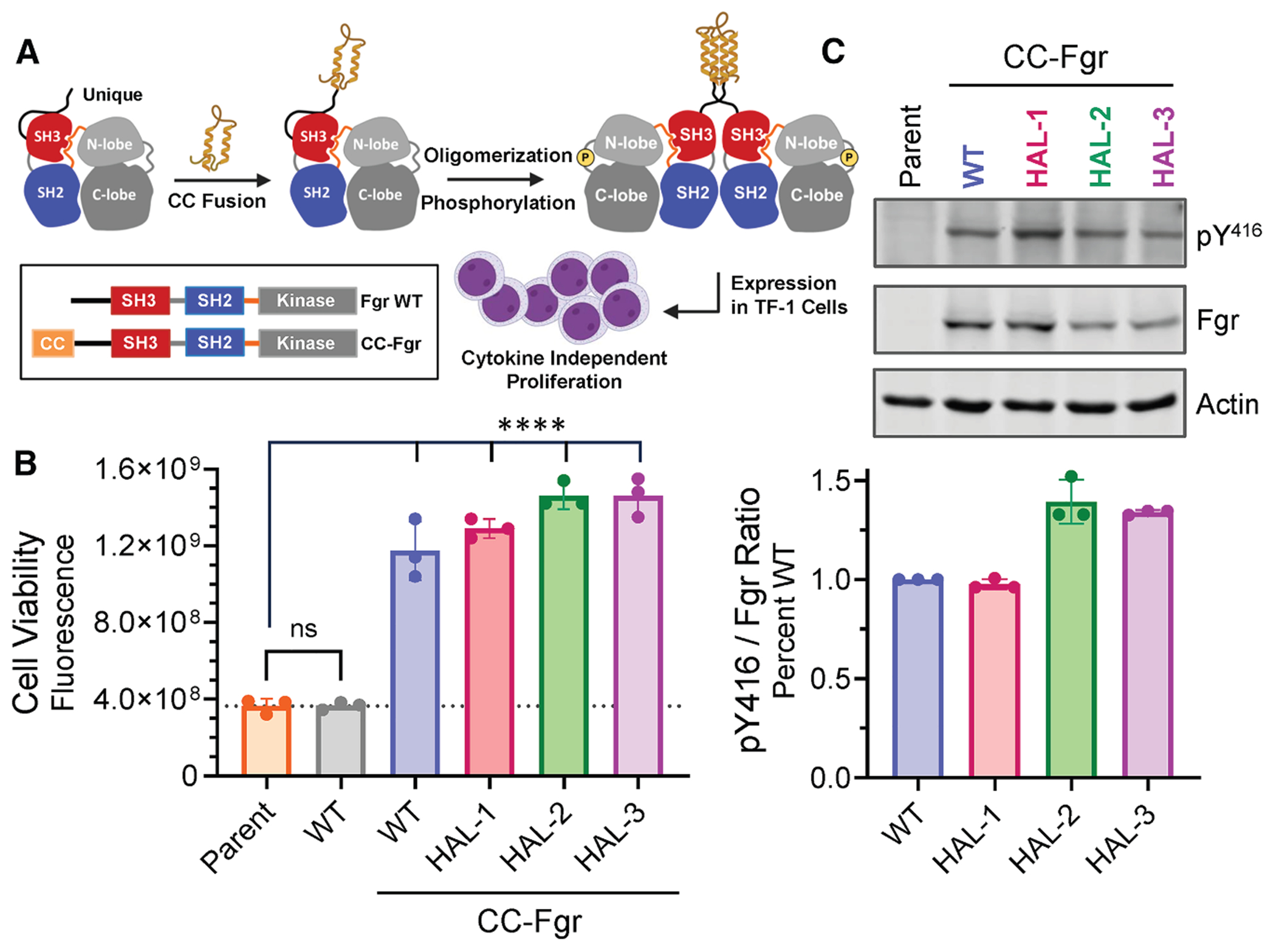
CC-Fgr HAL variants drive cytokine-independent growth of TF-1 myeloid leukemia cells (A) Fgr was activated by N-terminal fusion to the 70-amino acid CC domain of Bcr-Abl, promoting oligomerization and *trans*-phosphorylation. When expressed in TF-1 cells, the CC-Fgr fusion proteins drive GM-CSF-independent growth. (B) TF-1 erythroleukemia cells were transduced with empty vector (parent), WT Fgr or CC-fused Fgr proteins (WT and all three HAL variants). Cells were seeded at equal concentrations in the absence of GM-CSF and incubated at 37°C for 4 days. Cell viability was determined using the CellTiter-Blue assay which reports metabolic activity as fluorescence. Bar heights indicate the mean fluorescence ±SEM from three biological replicates. Groups were compared by one-way ANOVA relative to the parent cells; ****, *p* < 0.0001, ns, not significant. (C) Lysates of TF-1 cells expressing CC fusions of WT and HAL forms of Fgr were immunoblotted for kinase activation loop autophosphorylation (pY416) and kinase protein expression (Fgr) with actin as loading control. Immunoreactive bands were quantified with secondary antibodies tagged with infrared dyes and imaged using the LI-COR Odyssey platform. Parent TF-1 cell lysates were also analyzed as a negative control. Representative blots are shown at the top. Ratios of pTyr416 to kinase expression band intensities were determined for three independent replicates and normalized to WT and are shown in the bar graph as mean ± SEM. CC-Fgr HAL-2 and HAL-3 displayed a small (30%) increase in normalized autophosphorylation when compared to CC-Fgr with a WT linker.

Our next goal was to translate our observations with Fgr HAL proteins to a cell-based system with relevance for AML. To do so, we chose the TF-1 cell line as a model system. Derived from a human erythroleukemia case, these cells do not express endogenous Fgr or Hck and require the cytokine granulocyte-macrophage colony-stimulating factor (GM-CSF) to grow and survive in culture.^20^ Overexpression of WT Fgr alone is not sufficient to drive cytokine-independent growth in these cells.^4,14,20^ Therefore, we fused the N terminus of Fgr to the 70-amino-acid coiled-coil (CC) domain of the breakpoint cluster region protein associated with activation of the Abl tyrosine kinase in chronic myeloid leukemia (CML) (Figure 4A). The resulting CC-Fgr fusion protein was previously shown to drive cytokine-independent growth of TF-1 cells and render them sensitive to the SFK inhibitors A-419259 and TL02-59, demonstrating that the cells are now dependent on Fgr kinase activity to proliferate and survive.^20^ TF-1 cells expressing CC-Fgr proteins carrying each of the three HAL mutations also became cytokine-independent (Figure 4B), suggesting that enhanced SH3-linker interaction does not suppress signals for growth and survival in culture. Expression of WT and HAL forms of CC-Fgr proteins was confirmed by immunoblotting and all four proteins demonstrated autophosphorylation on the activation loop tyrosine (pY416; Figure 4C). HAL-2 and HAL-3 modification increased autophosphorylation by about 30% relative to the CC-Fgr control. Despite enhanced autophosphorylation, the HAL-3 variant showed reduced biological activity *in vivo* as described in more detail below.

**Figure 5.**
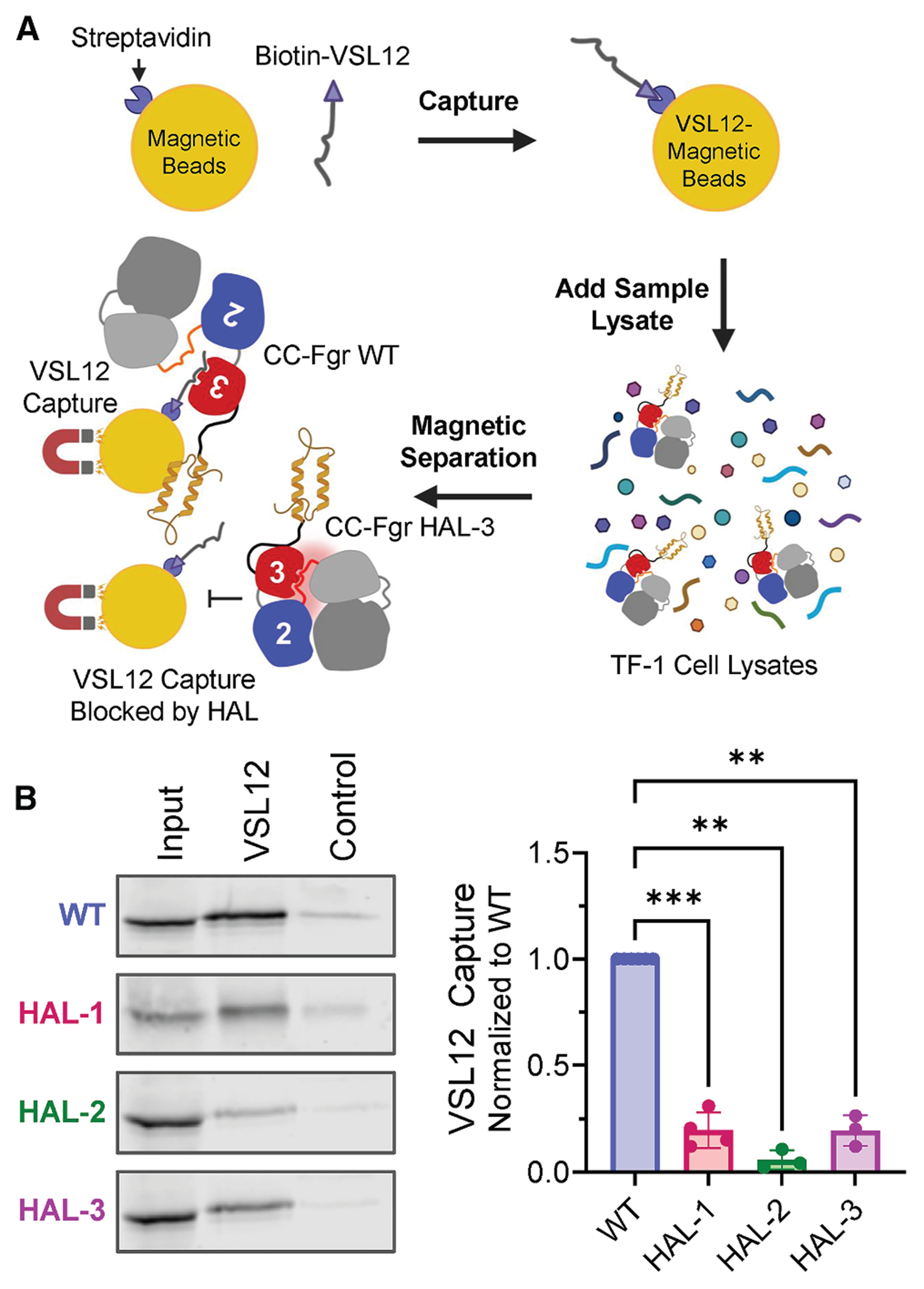
HALs in active Fgr fusion proteins maintain enhanced SH3 interaction in cells (A) Schematic diagram of the SH3 domain capture assay. Biotin-VSL12 peptides were immobilized on streptavidin-magnetic beads and incubated with lysates from TF-1 cells expressing CC-Fgr WT or HAL proteins. Beads were isolated by magnetic separation, washed and the level of associated Fgr proteins was assessed by immunoblotting. Fgr HAL proteins were predicted to demonstrate less capture due to enhanced intramolecular SH3-linker interaction. (B) VSL12 capture efficiency of Fgr proteins was determined by immunoblotting and quantified using the LICOR Odyssey imaging system. Representative blots (*left*) show the level of each Fgr protein in the lysate (Input), extent of Fgr protein capture (VSL12), and non-specific binding to SA-beads without the VLS12 peptide (Control). Quantification of the band intensities for three or four replicates from independent lysates is shown on the right. Non-specific binding was subtracted from the extent of Fgr protein capture and normalized to the level of input protein. Bar heights show the mean normalized band intensities ±SEM for at least three biological replicates and statistical comparison was performed by one-way ANOVA; **, *p* < 0.01, ***, *p* < 0.001.

To ensure that the HAL mutations retained enhanced SH3 domain engagement in the context of the CC-Fgr fusion protein in cells, we performed an SH3 domain capture assay. For these experiments, the SH3-binding peptide VSL12 was biotinylated and immobilized on streptavidin-coated magnetic beads and mixed with lysates from cells expressing the various forms of Fgr (Figure 5A). Following incubation and washing, associated kinase proteins were eluted and detected by immunoblotting. The degree of SH3 capture depends on conformational exposure of SH3. Using this assay, we recently showed that CC fusion increases exposure of the SH3 domain compared to WT Fgr, which may contribute to TF-1 cell transformation by enhancing downstream substrate engagement.^20^ Therefore, we reasoned that the HAL modifications may counteract the opening effect resulting from the CC fusion. Indeed, TF-1 cell lysates expressing CC-Fgr proteins with each of the HAL modifications displayed significantly lower capture by the VSL12 beads compared to the WT control (Figure 5B). These results show that CC-Fgr proteins bearing HALs retain SH3 domain occlusion in the cellular environment.

### Suppression of Fgr SH3 dynamics increases sensitivity to ATP-site inhibitors in AML cells

**Figure 6.**
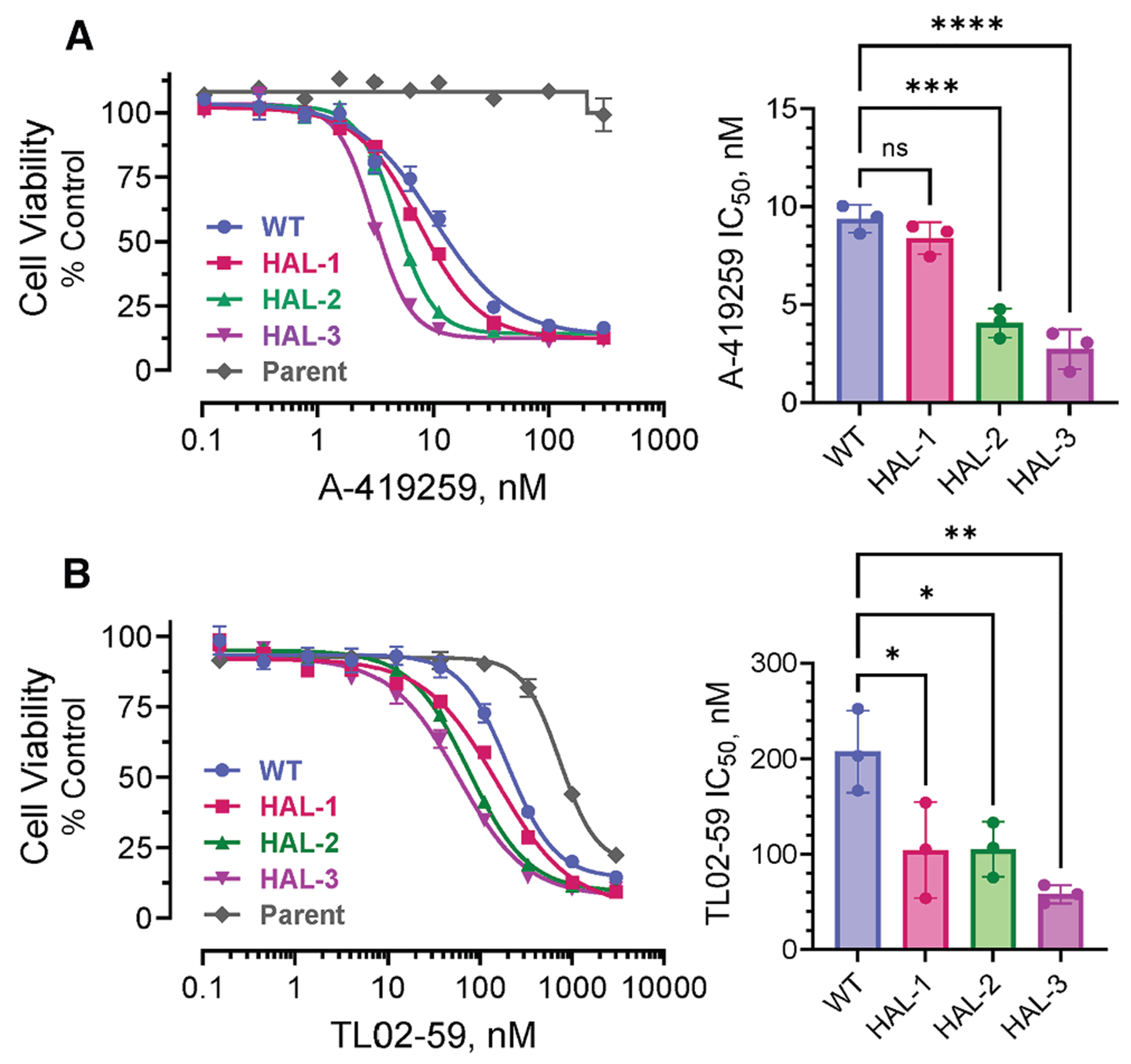
Suppression of Fgr SH3 dynamics increases sensitivity to ATP-site inhibitors in AML cells TF-1 cells expressing CC fusions of WT or HAL Fgr proteins were cultured in the presence of increasing concentrations of the ATP-site inhibitors A-419259 (A) and TL02-59 (B) and incubated at 37°C for 72 h. Cell viability was determined using the CellTiter-Blue assay and results normalized to control cultures treated with the carrier solvent alone (0.1% DMSO). Representative dose-response curves are shown on the left. IC_50_ values were determined for three biological replicates using non-linear curve fitting and are shown in the bar graphs on the right. Bar heights indicate the mean values ±SEM. Significant differences were determined by one-way ANOVA; *, *p* < 0.05, **, *p* < 0.01, ***, *p* < 0.001, ****, *p* < 0.0001, ns, not significant.

We previously showed that expression of CC-fused versions of both Fgr and Hck sensitized TF-1 cells to the ATP-site inhibitors A-419259 and TL02-59.^20^ Having verified that TF-1 cells expressing HAL versions of CC-Fgr maintained cytokine independent growth while retaining restricted regulatory domain dynamics, we tested whether these modifications affected sensitivity to these ATP-site inhibitors as observed with the recombinant Fgr-HAL3 protein *in vitro*. TF-1 cells expressing CC-Fgr fusions with and without the HAL modifications, as well as TF-1 parental cells grown in the presence of GM-CSF, were incubated over a range of inhibitor concentrations and cell viability was assessed 72 h later. TF-1 cells expressing HAL versions of CC-Fgr displayed increased sensitivity to both ATP-site inhibitors, with significant enhancement observed with A-419259 for HAL-2 and HAL-3 (Figure 6A) and all three HAL variants with TL02-59 (Figure 6B). These results suggest that both types of ATP-site inhibitors prefer a closed overall Fgr conformation in cells, consistent with the *in vitro* results.

### Suppression of Fgr SH3 domain dynamics significantly impairs bone marrow engraftment of TF-1 AML cells in a mouse model

**Figure 7.**
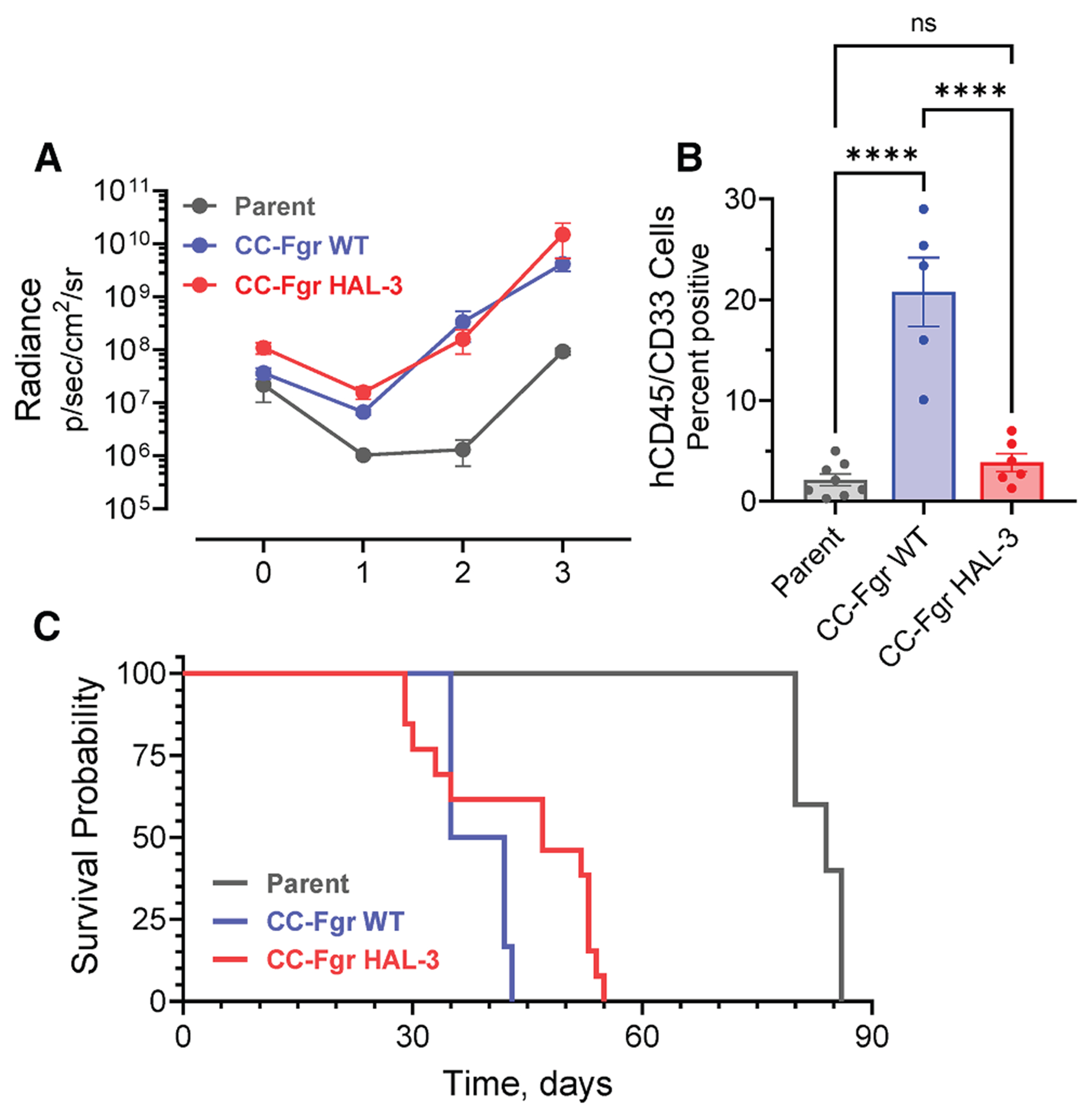
Suppression of Fgr SH3 domain dynamics significantly impairs bone marrow engraftment of TF-1 AML cells Immunocompromised (NSG) mice were injected with equal numbers of parental TF-1 cells (parent) or cells expressing active forms of WT Fgr (CC-Fgr WT) or the HAL-3 variant (CC-Fgr HAL-3; five to eight animals per group). Each cell population was tagged with luciferase to allow *in vivo* imaging of tumor growth. (A) Quantitative analysis of tumor growth measured over 3 weeks by *in vivo* image analysis of luciferase activity. Each data point represents the average radiance observed for all animals in each group ±SEM. (B) Four weeks after cell injection, mice were sacrificed, and bone marrow was analyzed for the presence of TF-1 cells by flow cytometry with antibodies specific to the human cell surface markers CD33 and CD45. Each dot represents one mouse. Bar heights indicate the average percentage of human cells present ±SEM; significance was assessed by one-way ANOVA (****, *p* < 0.0001; ns, not significant). (C) Kaplan-Meyer analysis of groups of mice injected with each cell line as indicated. Mice injected with CC-Fgr HAL-3 survived about 2 weeks longer than their WT counterparts, suggesting that the decrease in bone marrow engraftment due to the suppression of SH3 dynamics reduced Fgr leukemogenic potential.

Recently we demonstrated that expression of the active, CC-fused forms of Fgr and Hck promotes bone marrow engraftment of TF-1 cells in immunocompromised mice.^20^ To explore whether restriction of SH3 dynamics via HAL modifications impacts TF-1/CC-Fgr cell growth and bone marrow engraftment in this model, mice of the same immunocompromised strain were injected with equal numbers of parental TF-1 cells or cells expressing CC fusions of WT Fgr or the HAL-3 variant. Cells in each population were also tagged with a luciferase reporter, allowing us to track overall tumor burden in the live animals over time using an *in vivo* imaging system. Mice injected with cells expressing either the WT or HAL-3 version of CC-Fgr showed similar levels of overall tumor burden by *in vivo* imaging with levels significantly higher than those observed with the parental cells (Figure 7A). At the end of 4 weeks, mice in each group were sacrificed and the percentage of human AML cells present in the bone marrow was assessed by flow cytometry. Mice injected with TF-1 cells expressing CC-Fgr with the WT linker showed significantly higher bone marrow engraftment compared to parental TF-1 cells, consistent with our previous work.^20^ Remarkably, this effect was completely reversed in bone marrow from mice injected with TF-1/CC-Fgr-HAL-3 cells, with engraftment statistically indistinguishable from the control group injected with parental TF-1 cells (Figure 7B). Kaplan-Meyer analysis shows that mice injected with CC-Fgr HAL-3 survived about 2 weeks longer than their CC-Fgr WT counterparts, suggesting that the decrease in bone marrow engraftment due to the suppression of SH3 dynamics reduced Fgr leukemogenic potential (Figure 7C).

## DISCUSSION

In the present study, we engineered modified versions of Fgr predicted to favor a closed regulatory conformation, allowing us to investigate how regulatory domain dynamics influence ATP-site inhibitor potency and downstream signaling in the context of AML cells. Structure-guided substitutions in the SH2-kinase linker (T252P and A256R) were designed to stabilize the polyproline type II (PPII) helical character of the linker and introduce a new salt bridge with the SH3 domain. Using X-ray crystallography, fluorescence polarization, and SPR, we demonstrate that these HAL substitutions strengthen intramolecular SH3-linker interaction.

Using a recombinant Fgr construct containing both substitutions (HAL-3), we found that enhanced SH3-linker interaction increased the apparent K_m_ for ATP in kinetic assays. This observation was unexpected given our previous results suggesting that Fgr catalytic activity is relatively insensitive to disruption of SH3-SH2 regulatory domain interactions.^14^ In that earlier work, downregulated forms of Fgr, Src, and Hck were tested for activation by peptides that displace regulatory domain contacts, including the SH3-binding peptide VSL12. Whereas Src and Hck were readily activated by SH3 or SH2 domain displacement, Fgr activity remained unchanged despite clear peptide binding measured by SPR. These results suggested that regulatory domain displacement alone is insufficient to modulate Fgr catalytic activity under these conditions. In contrast, the HAL substitutions described here strengthen SH3-linker interaction and stabilize the closed regulatory configuration, revealing that sufficiently strong regulatory domain interactions can allosterically influence Fgr catalytic properties. Together, these observations suggest that while Fgr may be less sensitive than other SFKs to regulatory domain displacement, stabilization of these interactions can still reshape the catalytic environment of the kinase domain. This finding raises the possibility that enhancement of SH3-linker interaction with a small molecule could provide a route to allosteric control of Fgr activity.

Restriction of Fgr regulatory domain dynamics through enhanced SH3-linker interaction increased the apparent potency of TL02-59 and A-419259 in biochemical assays as well as in TF-1 cells transformed by active CC-Fgr HAL variants. These observations suggest that both ATP-competitive inhibitors preferentially engage a closed conformation of Fgr in which the SH3 domain remains bound to the linker. This finding was somewhat unexpected given the distinct binding modes of these inhibitors and their opposing effects on Fgr conformation observed in crystal structures and hydrogen-deuterium exchange mass spectrometry experiments.^12^ Enhanced SH3-linker interaction also increased the K_m_ of Fgr for ATP, which likely contributes to the observed enhancement of sensitivity to ATP-competitive inhibitors. These findings parallel earlier work with Bcr-Abl, the oncogenic tyrosine kinase associated with CML. The Abl kinase domain contains a Src-like SH3-SH2-kinase core module in which SH3-linker interaction contributes to negative regulation of activity.^21^ Introduction of proline substitutions within the linker enhanced SH3 binding in recombinant Abl core proteins and Bcr-Abl, resulting in increased sensitivity to both the type II ATP-site inhibitor imatinib and the allosteric inhibitor GNF-2.^22^ The latter compound binds a deep pocket in the C-lobe of the Abl kinase domain and ultimately led to development of the clinically approved Bcr-Abl inhibitor asciminib. Importantly, combining ATP-site inhibitors with asciminib suppresses the emergence of drug-resistant CML cells in animal models and clinical studies.^23–25^

Recent work has further highlighted the importance of conformational dynamics in regulating SFK activity. Using solution NMR spectroscopy, a transient intermediate state was identified within the conformational landscape of the Src kinase domain that lies between canonical active and inactive conformations and promotes rapid ADP release following ATP hydrolysis.^26^ This intermediate state enables efficient catalytic turnover and supports processive phosphorylation of multisite substrates, a defining feature of some SFK signaling. Perturbation of this state impaired processive phosphorylation by multiple family members, including Src, Lck, and Hck, demonstrating that dynamic sampling of alternative conformations is critical for efficient signaling by these kinases. These findings emphasize that SFKs operate within a complex conformational landscape in which transient structural states influence catalytic efficiency and signaling output. In this context, our observation that stabilization of SH3-linker interaction alters ATP affinity and inhibitor sensitivity in Fgr supports the broader concept that perturbations to the SFK conformational landscape, whether through regulatory domain interactions (as observed previously with the Abl SH3-SH2-kinase core^27^) or small-molecule binding,^19,28^ can reshape catalytic behavior and pharmacologic response.

Finally, we observed that TF-1 AML cells expressing SH3-restricted forms of Fgr displayed reduced leukemogenic potential following transplantation into immunocompromised mice. Mice injected with cells expressing WT or HAL-3 CC-Fgr showed similar overall tumor burden by whole-animal imaging, consistent with comparable proliferation in liquid culture. However, TF-1 cells expressing CC-Fgr HAL-3 exhibited significantly reduced bone marrow engraftment compared with cells expressing WT CC-Fgr. These findings suggest the possibility that accessibility of the SH3 domain may be required for signaling events that support survival within the bone marrow microenvironment. Accordingly, small-molecule SH3 antagonists may interfere with Fgr-dependent signaling in a manner analogous to the HAL-3 modification in this anatomical context. In recent work, we showed that both CC fusion and the gatekeeper mutation T338M activate Fgr and enhance bone marrow engraftment by TF-1 cells.^20^ Future studies will determine whether enhanced SH3-linker interaction can similarly attenuate the transforming potential of the Fgr gatekeeper mutant as well as analogous active forms of Hck.^20^

### Limitations of the study

Although our structural and biophysical data support a model in which HAL substitutions stabilize a closed Fgr conformation, aspects of this mechanism are inferred indirectly. Crystal structures of the regulatory domains lack the kinase domain and crystal packing effects limited direct visualization of SH3-linker engagement in the HAL-2 and HAL-3 variants. In addition, our use of engineered CC-Fgr fusion proteins and TF-1 cells may not fully recapitulate native regulatory and signaling contexts in primary AML cells, which often express multiple active Src-family members^4^ as well as other non-receptor tyrosine kinases including Fes^29^ and Syk.^30^ While restriction of regulatory domain dynamics enhances ATP-site inhibitor sensitivity *in vitro* and in cells, the relationship between these effects and *in vivo* phenotypes is complex. A key unanswered question relates to changes in downstream substrates and signaling events that may result from restricted SH3 domain access. *In vitro*, TF-1 cells expressing WT or HAL forms of CC-Fgr grow equally well in the absence of GM-CSF and show essentially equal autophosphorylation (Figure 4), suggesting that the HAL substitutions are not sufficient to suppress the kinase activity of CC-Fgr per se. Bulk growth of the two populations of cells in mice is also the same, as demonstrated by *in vivo* imaging (Figure 7). The difference in cellular behavior is limited to the bone marrow environment, suggesting the possibility that cellular engraftment of that niche requires WT Fgr SH3 domain access to enable cellular survival. Thus, exploring signaling differences between the two cell populations in liquid culture may not explain what is observed in bone marrow. Instead, what may be required is assessment of differences in signaling in the bone marrow environment, possibly by spatial transcriptomics or proteomics.

## RESOURCE AVAILABILITY

### Lead contact

Requests for further information and resources should be directed to and will be fulfilled by the lead contact, Thomas E. Smithgall (tsmithga@pitt.edu).

### Materials availability

All unique reagents generated in this study are available from the lead contact with a completed materials transfer agreement and subject to availability.

## STAR★METHODS

### EXPERIMENTAL MODEL AND STUDY PARTICIPANT DETAILS

#### Bacterial strains

Routine subcloning was performed in *E. coli* strain DH5α. Expression of recombinant Fgr proteins for use in biochemical and biophysical assays as well as X-ray crystallography were expressed in *E. coli* strain BL21 (DE3) pLysS.

#### Human cell lines

Recombinant retroviral vectors for gene transfer were generated in transfected 293T cells which are of human female embryonic kidney origin. 293T cells were cultured in DMEM supplemented with 10% FBS. The TF-1 erythroblast cell line was isolated from the bone marrow of a 35-year-old Asian male with severe pancytopenia in 1987. These cells were cultured in RPMI 1640 medium supplemented with 10% FBS, 2 mM L-glutamine, and Antibiotic–Antimycotic. Parental TF-1 cells require culture medium supplemented with 100 ng/mL human GM-CSF. All mammalian cells were cultured in humidified 37°C incubators with a 5% CO_2_ atmosphere. Both cell lines were obtained from the American Type Culture Collection (ATCC) and have been authenticated by the ATCC through short tandem repeat (STR) profiling and tested for mycoplasma contamination.

#### Animal studies

Male and female NOD.Cg-*Prkdc^scid^ Il2rg^tm1Wjl^*/SzJ (NSG) immunodeficient mice, typically 6 to 12 weeks of age, were used for the human AML cell bone marrow engraftment studies. Mice were purchased from Jackson Laboratories. All experimental protocols involving engraftment of immunocompromised mice with human AML cells were reviewed and approved by the University of Pittsburgh Institutional Animal Care and Use Committee. All animal experiments conform to the required institutional regulatory standards.

### METHOD DETAILS

#### Cloning, expression and purification of recombinant Fgr regulatory domain proteins

Human Fgr regulatory domain coding sequences (SH3-SH2 and SH3-SH2-linker) were PCR amplified and subcloned into a bacterial expression vector that allows expression from a T7 promoter (pET28a). All sequences were cloned downstream and in-frame with a His6-Smt3 (yeast SUMO) tag sequence using standard restriction digestion cloning protocols, resulting in plasmids encoding the Fgr regulatory domains with an N-terminal His6-Smt3 solubility/purification tag. HAL linker substitutions were incorporated into the coding sequence using the QuickChange II site directed mutagenesis kit (Agilent). The complete nucleotide sequences of all Fgr coding regions were confirmed by Sanger DNA sequencing. For bacterial expression of Fgr regulatory domain proteins, One Shot BL21(DE3)pLysS chemically competent *E. coli* cells (Thermo Fisher) were transformed with Fgr expression plasmids using the manufacturer’s instructions. Transformed cells were grown at 37°C with 250 rpm shaking to an OD_600_ of 0.6 - 0.8 in 1 L of Terrific Broth containing 30 μg/mL kanamycin. The incubation temperature was then reduced to 16°C and protein expression was induced by the addition of IPTG to a final concentration of 0.25 mM. After 18 h of induction, the cells were harvested via centrifugation and either immediately lysed for protein purification or snap frozen in liquid nitrogen and stored at −80°C. For purification, pellets from 1 L cultures were resuspended in Ni-IMAC binding buffer (25 mM Tris-HCl, pH 8.2, 500 mM NaCl, 20 mM imidazole, 2 mM β-mercaptoethanol (BME), and 10% glycerol) supplemented with a cOmplete EDTA-free protease inhibitor cocktail tablet (Millipore Sigma) prior to lysis using a microfluidizer. The soluble protein fraction was isolated by ultracentrifugation at 100,000 g for 1 h at 4°C. Clarified lysates were loaded onto a 5 mL HisTrap HP column (Cytiva) and eluted with a linear gradient using Ni-IMAC elution buffer (25 mM Tris-HCl, pH 8.2, 500 mM NaCl, 500 mM imidazole, 2 mM BME, and 10% glycerol). Fractions containing the recombinant proteins were pooled and dialyzed against Ni-IMAC dialysis buffer (25 mM Tris-HCl, pH 8.2, 150 mM NaCl, 2 mM BME, and 10% glycerol) at 4°C overnight. The His6-Smt3 tag was cleaved by the addition of the His6-ULP1 SUMO protease (0.2 mL of a 2 mg/mL stock) to the dialyzed Fgr protein and incubation with rocking at 4°C for 1 h. After cleavage, the Fgr protein was separated from the SUMO tag by loading the sample onto a 5 mL HisTrap HP column (Cytiva) as above for the first Ni-IMAC step; purified untagged Fgr protein was collected in the flow-through fraction. The NaCl concentration was lowered to 25 mM and protein was loaded onto a 5 mL HiTrap Q HP anion exchange column (Cytiva) followed by gradient elution with ion exchange elution buffer (25 mM Tris-HCl, pH 8.2, 1.0 M NaCl, 2 mM BME, and 10% glycerol). For the final step proteins were dialyzed against size-exclusion chromatography (SEC) buffer (25 mM Tris-HCl, pH 8.3, 150 mM NaCl, 10% glycerol, and 2 mM BME), concentrated to 5.0 mL and run over a HiLoad 16/600 Superdex 200 preparative SEC column (Cytiva). Fractions containing purified Fgr proteins were pooled, concentrated to ~5 mg/mL and stored at −80°C until use. None of the HAL mutations affected the protein purification process. The purity and integrity of each protein was verified after each step using SDS-PAGE and Coomassie blue protein staining.

#### Expression and purification of recombinant near full-length Fgr WT and HAL-3

The near-full-length Fgr coding sequence (human isoform, amino acids 80–523 with an N-terminal His6-tag and modified C-terminal tail encoding YEEIP) was PCR-amplified and subcloned into a pET-28a bacterial expression vector (Millipore Sigma). HAL-3 mutations were introduced using the QuickChange II site directed mutagenesis kit (Agilent). The expression plasmids were used to transform BL21(DE3)pLysS *E. coli* (Thermo Fisher) using the manufacturer’s instructions. To facilitate soluble expression of the near full-length proteins in the closed conformation, the cells were co-transformed with a pET-DUET plasmid encoding both the human PTP1B tyrosine phosphatase catalytic domain (amino acids 1–283) and full-length Csk tyrosine kinase behind T7 promoters.^11,31^ Additionally, to improve solubility, cells were co-transformed with the pACYC-Skp-GroEL/ES plasmid (Addgene) to express cytoplasmic copies of the Skp and GroEL/ES chaperones. Soluble Fgr was then purified by sequential Ni-IMAC affinity, size exclusion, and anion exchange chromatography steps as described in detail elsewhere.^31^

#### Fluorescence polarization

The Src-family kinase SH3-binding peptide, VSL12 (VSLARRPLPPLP), was synthesized and labeled with 6-carboxyfluorescein at its N terminus (University of Pittsburgh Peptide Synthesis Core Laboratory). The molecular weight and purity of the peptide were verified by mass spectrometry. VSL12 stock solutions (1.0 mM) were prepared in FP assay buffer (20 mM Tris-HCl, pH 8.3, 1% DMSO). FP assays were performed in low-volume black 384-well plates with a non-binding surface (Corning). VSL12 peptide (1.25 μM) and increasing concentrations of Fgr proteins were added to each well in FP assay buffer for a final assay volume of 20 μL and mixed by shaking for 5 min at room temperature. The average FP signals derived from ten flashes per well were recorded at an excitation wavelength of 485 nm and emission wavelength of 515 nm using a Molecular Devices microplate reader and the Softmax Pro software.

#### *In vitro* kinase assays

The activities of near-full-length recombinant Fgr kinases (wild-type vs. HAL-3 variant) were measured using the ADP Quest kinetic kinase assay (DiscoverX/Eurofins) which fluorometrically follows the production of ADP.^32^ Kinase reactions (50 μL) were performed in low-volume black 384-well plates with a non-binding surface (Corning) in kinase assay buffer (15 mM HEPES, pH 7.4, 20 mM NaCl, 1 mM EGTA, 0.02% Tween 20, 10 mM MgCl_2_, and 0.1 mg/mL bovine γ-globulins). To determine the apparent K_m_ for ATP, Fgr proteins were assayed at 150 ng/well with ATP titrated by 1:1 serial dilution starting at 100 μM and a fixed concentration of the substrate peptide, YIYGSFK (500 μM). To determine IC_50_ values of ATP-site inhibitors, A-419259 and TL02–59, the ATP concentration (100 μM) was set in excess of the K_m_ while keeping the same protein and substrate concentrations. Both inhibitors were solubilized in kinase assay buffer. Compounds were added to the Fgr proteins in the presence of all other assay components except for ATP. The assay plate was incubated for 15 min at room temperature to allow for ATP-site inhibitor binding followed by addition of ATP to start the kinase reactions. Fluorescence was measured every 5 min for 3 h using a SpectraMax i3x plate reader (Molecular Devices). The fluorescence values for each condition were averaged across four technical replicates. For each compound concentration, the linear portion of the reaction progress curve was fit by regression analysis (GraphPad Prism). The slope of the line and the calculated SEM for each concentration were then graphed as a function of compound concentration followed by nonlinear regression analysis to calculate IC_50_ values. Fluorescence values were converted to pmol ADP using a standard curve of known ADP concentrations.

#### Crystallization

Purified recombinant wild-type Fgr SH3-SH2-linker and Fgr SH3-SH2-HAL proteins were crystallized from starting concentrations of 0.75, 3.2, 4.0, and 8.1 mg/mL for wild-type and HALs 1, 2 and 3, respectively. Crystals were grown via sitting-drop vapor diffusion at room temperature by mixing each protein solution with the following mother liquors in a 1:1 ratio: wild-type Fgr, 0.2 M sodium chloride, 0.1 M Bis-Tris, pH 5.5, 25% (w/v) PEG 3350; Fgr HAL-1, 0.2 M sodium chloride, 0.1 M MES monohydrate, pH 6.0, 20% (w/v) polyethylene glycol 6,000; Fgr HAL-2, 0.1 M trisodium citrate pH 5.5, 2.5 M ammonium sulfate; Fgr HAL-3, 0.2 M sodium chloride, 0.1 M Bis-Tris, pH 5.5, 25% (w/v) PEG 3350. Crystals grew in about 1 week and were cryoprotected using 30% (v/v) glycerol in the mother liquor followed by flash freezing in liquid nitrogen.

#### X-Ray data collection, processing and structure determination

X-ray data were collected at the 23-ID-B and 23-ID-D beamlines at the Advanced Photon Source, Argonne National Laboratory, U.S. Department of Energy. The crystals diffracted to a resolution of 1.8 Å (wild-type), 1.3 Å (HAL-1), 1.8 Å (HAL-2), and 1.6 Å (HAL-3). Data indexing, integration, and scaling were conducted using the XDS, Xia2-Dials and Aimless program suites.^33–35^ Phasing and structure solution were performed by molecular replacement with the program PHASER.^36^ The individual SH3 and SH2 domains from the near-full-length Fgr structure (PDB ID: 7UY0) were used as independent search models. For molecular replacement, the SH2-kinase linker was not included in the search model prior to phasing due to its flexible nature. Iterative molecular replacement used the found solutions as fixed models in combination with search models. The molecular replacement solution was refined using rigid body, simulated annealing (phenix.refine), restrained, individual isotropic B-factor, occupancy and TLS refinement using Refmac5.^37,38^ Rounds of refinement and model building were conducted using Refmac5^39^ and Coot,^40^ respectively. Water molecules were added using Refmac5, and additional water and solvent atoms were added using Coot. Final models of the X-ray structures presented in the figures were produced using PyMol (Schrödinger). All structures were refined further using the Phenix^38,41^ and CCP4^42^ software suites. X-ray data and final refinement statistics are summarized in Table S1.

#### Surface plasmon resonance

SPR analysis was conducted using a Reichert 4SPR instrument (Reichert Technologies). Near full-length Fgr wild type, near full-length Fgr HAL-3 and Fgr SH3-SH2 proteins were immobilized on carboxymethyl dextran hydrogel biosensor chips (Reichert) using standard amine coupling chemistry with 1-ethyl-3-(-3-dimethylaminopropyl) carbodiimide hydrochloride (EDC) and N-hydroxysuccinimide (NHS). The SH3-binding peptide VSL12 was injected in SPR buffer (10 mM HEPES, pH 7.4, 150 mM NaCl, 0.05% Tween 20, and 3 mM EDTA) over a range of concentrations at 50 μL/min with a 90 s association phase and 120 s dissociation phase. The chip surface was regenerated with 1 mM NaOH after each peptide injection. Each concentration of VSL12 was measured in quadruplicate, and the resulting sensorgrams were corrected for buffer effects. Kinetic and binding constants were calculated by fitting the sensorgrams using the 1:1 Langmuir binding model in the TraceDrawer program (Reichert).

#### Expression vector construction and retroviral gene transfer

Coding sequences for human Fgr were subcloned into the retroviral vector, pMSCVpuro (Takara) as described elsewhere.^20^ To create CC-Fgr, the coding sequence for the 70 amino acid coiled-coil domain of Bcr was amplified by PCR and subcloned in frame to the 5′ end of the Fgr cDNA. The HAL substitutions were then introduced using the QuikChange II site-directed mutagenesis system (Agilent). The complete nucleotide sequences of all Fgr coding regions were confirmed prior to use. Recombinant retroviruses were produced from the pMSCVpuro vectors in 293T cells and used to transduce TF-1 cells as described.^20^

To evaluate Fgr protein expression and activation loop phosphorylation, TF-1 cells were washed with cold PBS and resuspended in Cell Lysis Buffer (20 mM Tris-HCl, pH 7.5, 150 mM NaCl, 1 mM EDTA, 1 mM EGTA, 1% Triton, 2.5 mM sodium pyrophosphate, 1 mM β-glycerophosphate, 1 mM Na_3_VO_4_, 1 μg/mL leupeptin; Cell Signaling Technology) supplemented with cOmplete protease inhibitor cocktail (Millipore Sigma). Following sonication, lysates were clarified by microcentrifugation at 4°C and soluble protein concentrations were determined using the Bio-Rad Protein Assay Dye Reagent. Lysate proteins were separated by SDS-PAGE, transferred to nitrocellulose membranes, and probed with the following primary antibodies: Fgr rabbit polyclonal Ab (Cell Signaling Technologies); Src pY416 (Millipore Sigma); Actin mouse mAb (Millipore Sigma). Immunoreactive proteins were visualized using secondary antibodies conjugated to infrared dyes (LI-COR) and scanned on an LI-COR Odyssey CLx imaging system.

#### TF-1 cell viability assay

TF-1 cell populations were seeded at 100,000 cells/mL in 6-well culture plates in the presence and absence of GM-CSF. Cell viability was assessed every 24 h over four days using the CellTiter-Blue Cell Viability assay (Promega) according to the manufacturer’s protocol. Fluorescence intensity was measured with an excitation wavelength of 560 nM and emission wavelength of 590 nm.

#### Kinase inhibitor sensitivity assays

TL02-59 was reconstituted to 10 mM in DMSO and diluted into cell cultures with a final DMSO concentration of 0.1%. A-419259 was reconstituted as a 10 mM stock in autoclaved Milli-Q water. TF-1 cells were seeded at 100,000 cells per mL in 96-well culture plates, and serial dilutions of each inhibitor were added to triplicate wells. Cell viability was assessed 72 h later using the CellTiter-Blue Cell Viability assay described above. IC_50_ values were determined by non-linear regression analysis of concentration-response curves with the GraphPad Prism software package.

#### SH3 domain capture assay

The SH3 domain capture assay for open conformations of Src family kinases in solution is described elsewhere.^20^ In short, biotinylated VSL12 peptide was immobilized on streptavidin (SA) Dynabeads (ThermoFisher). VSL12-SA-Dynabeads were resuspended in 0.5 mL PBS plus 0.01% BSA and placed on ice. Control SA-Dynabeads without VSL12 were similarly prepared. Clarified TF-1 cell lysates were prepared as described above and soluble protein (0.5–2.0 mg) was diluted to 1.0 mL in Cell Lysis buffer and combined with 20 μL of the VSL12-SA-Dynabead prep or no peptide control beads, followed by incubation for 1 h at 4°C. Beads were washed 3 times with 1.0 mL PBS supplemented with 0.1% Tween 20, resuspended in 20 μL 2× SDS sample buffer, and heated at 95°C for 5 min. Bound Fgr proteins were detected by immunoblotting as described above.

#### Mouse AML Xenograft Model

For tumor burden studies, 6-12-week-old male and female NSG mice were injected with busulfan (25 mg/kg; Millipore Sigma) intraperitoneally once a day for two days, and the following day injected with TF-1 cell populations suspended in PBS via the tail vein (5 × 10^6^ cells/mouse). *In vivo* imaging of bulk tumor cell growth in the mice was performed under isoflurane anesthesia. Each mouse was injected with 165 mg/kg of luciferin (Revvity) intraperitoneally right after cell injection and weekly until the end of the 4th week. Photons were detected using a Lumina S5 (Revvity) *in vivo* imaging system (IVIS) and analyzed using Living Image software (Revvity) and GraphPad Prism. At the end of the fourth week, all mice were euthanized by CO_2_ exposure and bone marrow was collected for flow cytometry with antibodies to human cell surface CD33 and CD45 (BD Biosciences). For survival studies, 6-12-week-old mice were injected with cells and tumor burden was monitored by IVIS weekly until mice succumbed from the tumor burden or met euthanasia guidelines.

#### Flow cytometry

Bone marrow samples from engrafted mice were analyzed for TF-1 cells by flow cytometry with human CD45-FITC and human CD33-APC antibody conjugates (BD Biosciences). Red blood cells in the bone marrow were lysed in 10 mM KHCO_3_, pH 7.4, 155 mM NH_4_Cl, and 130 μM EDTA prior to antibody staining as described.^16^ Cells were analyzed on a BD Accuri C6 Plus flow cytometer, and the resulting data were evaluated using BD CSampler Plus software and GraphPad Prism.

### QUANTIFICATION AND STATISTICAL ANALYSIS

Statistical details of all experiments are presented in the figure legends. Fluorescence polarization experiments were performed with four technical replicates per condition with each data point representing the mean ± SEM. Statistical significance was assessed by one-way ANOVA. SPR studies were performed with each peptide concentration assayed in triplicate and all data were integrated to determine the rate constants for association and dissociation. Curves were fit to a 1:1 Langmuir model using the TraceDrawer software package (Reichert). Two independent SPR trials were performed, and the resulting data are presented separately in Figure S2. Kinase assays were performed with three independent biochemical replicates, and each data point was assessed with quadruplicate technical replicates. Data are presented as mean values ±SEM and statistical differences between pairs were assessed using Student’s *t* test. Cell proliferation assays were determined with three biological replicates per condition. Mean values are presented ±SEM and significant differences assessed via one-way ANOVA. Fgr protein autophosphorylation in cell lysates and interaction with VSL12 beads were assessed with at least triplicate biological replicates and quantified using Licor infrared imaging. Mean band intensities are shown ±SEM and tested for significance by one-way ANOVA in the case of the SH3-binding assay. Inhibitor sensitivity assays both in cells and *in vitro* were performed over a range of compound concentrations and results best-fit by non-linear regression analyses. IC50 values were estimated from three biological replicates and significance between groups assessed by one-way ANOVA. Mouse studies were performed with 5–8 animals per group and *in vivo* imaging and flow cytometry results from each animal are presented as the mean ± SEM with statistical differences determined via one-way ANOVA. All statistical analyses were performed with GraphPad Prism, version 10.6.1. X-ray data collection and refinement statistics for each crystal structure are presented in Table S1.

## Supplementary Material

1

Supplementary data related to this article can be found online at https://doi.org/10.1016/j.celrep.2026.117551.

## Figures and Tables

**Table:** KEY RESOURCES TABLE

REAGENT or RESOURCE	SOURCE	IDENTIFIER
Antibodies		
Fgr rabbit polyclonal Ab	Cell Signaling Technologies	# 2755; RRID:AB_2246957
Src pY416 monoclonal Ab	Millipore Sigma	# 05-677; RRID:AB_309898
Mouse monoclonal anti-Actin, clone C4	Millipore Sigma	# MAB1501R; RRID:AB_2223041
IRDye 680LT Donkey anti-Mouse IgG	LI-COR	# 926-68022: RRID:AB_10715072
IRDye 800CW donkey anti-rabbit Ab	LI-COR	# 926-32213; RRID:AB_621848
Human anti-CD45-FITC conjugate	BD Biosciences	# 555482, RRID:AB_395874
Human anti-CD33-APC conjugate	BD Biosciences	# 340474, RRID:AB_400518
Bacterial and virus strains		
*E. coli* DH5α	Thermo Fisher	# 18265017
*E. coli* BL21 (DE3) pLysS	Thermo Fisher	# C602003
Chemicals, peptides, and recombinant proteins		
Puromycin	Millipore Sigma	# P9620
Cell Lysis Buffer	Cell Signaling Technologies	# 9803
Antibiotic-Antimycotic	Life Technologies	# 15240062
Protease Inhibitor Cocktail	Millipore Sigma	# 11697498001
Human GM-CSF	Life Technologies	# 15240062
VSL12 SH3-binding peptide	U. Pittsburgh Peptide Core	Custom synthesis
YIYGSFK Fgr substrate peptide	U. Pittsburgh Peptide Core	Custom synthesis
A-419259 kinase inhibitor	Cayman Chemical	#18168
TL02-59 kinase inhibitor	A Chem Tek	Custom synthesis
IVISbrite D-Luciferin Bioluminescent Substrate	Revvity	# 770504
Streptavidin M-270 Dynabeads	Thermo Fisher	# 65305
Critical commercial assays		
ADP Quest kinetic kinase assay	Eurofins/DiscoverX	# 90-0071
CellTiter-Blue cell viability assay	Promega	# G8080
QuikChange II site directed mutagenesis kit	Agilent	# 200523
Deposited data		
Fgr SH3-SH2-linker, WT crystal structure	This study	PDB: 10DT
Fgr SH3-SH2-HAL1 crystal structure	This study	PDB: 10FS
Fgr SH3-SH2-HAL2 crystal structure	This study	PDB: 10FV
Fgr SH3-SH2-HAL3 crystal structure	This study	PDB: 10GA
Experimental models: Cell lines		
TF-1 Cells	ATCC	# CRL-2003
293T Cells	ATCC	# CRL-3216
Experimental models: Organisms		
NOD.Cg-*Prkdc^scid^ Il2rg^tm1Wjl^*/SzJ (NSG) immunodeficient mice	Jackson Laboratories	# 005557
Recombinant DNA		
pET-28a bacterial expression plasmid	Millipore Sigma	# 69864
pET-DUET bacterial expression vector	Millipore Sigma	# 71146
pACYC-Skp-GroEL/ES expression vector	Addgene	# 83922
pMSCVpuro retroviral vector	Takara	# 634401
Software and algorithms		
Prism for statistical analysis, v 10.1	GraphPad	https://www.graphpad.com/
TraceDrawer for SPR curve fitting	TraceDrawer	https://tracedrawer.com/
Living Image for *in vivo* imaging	Revvity	https://www.revvity.com/software-downloads
Image Studio for immunoblot quantitation	LI-COR Biosciences	https://www.licor.com/bio/image-studio/
PyMol for molecular modeling	Schrödinger	https://www.pymol.org/
BioRender for illustrations	BioRender	https://www.biorender.com/
Other		
HisTrap HP column	Cytiva	# 17524802
HiLoad 16/600 Superdex 200 prep grade column	Cytiva	# 28989333
Reichert 4SPR Instrument	Reichert Technologies	https://www.reichertspr.com/en/products/reichert-4spr
SpectraMax i3x plate reader	Molecular Devices	https://www.moleculardevices.com/products/microplate-readers/multi-mode-readers/spectramax-i3x-readers
Carboxymethyl dextran SPR chips	Reichert Technologies	# 13206066
LI-COR Odyssey CLx Infrared imager for quantitative immunoblot analysis	LI-COR Biosciences	https://www.licor.com/bio/odyssey-dlx/
BD Accuri C6 plus flow cytometer	Waters Biosciences	https://www.bdbiosciences.com/en-us/products/instruments/flow-cytometers/research-cell-analyzers/bd-accuri-c6-plus

## Data Availability

Crystallographic coordinates for the four X-ray crystal structures of the Fgr SH3-SH2-linker proteins presented in this study have been deposited at the RCSB Protein Data Bank and will be made publicly available as of the date of publication. The PDB identifiers are as follows: wild-type, PDB: 10DT; HAL-1, PDB: 10FS; HAL-2, PDB: 10FV; HAL-3, PDB: 10GA. All other data presented in this study will be shared by the lead contact upon request. This study does not report original code. Any additional information required to reanalyze the data reported in this study is available from the lead contact upon request.

## Highlights

- SH3 and SH2 domains influence Src-family kinase signaling and inhibitor sensitivity
- Enhancing internal Fgr SH3-linker interaction constrains conformational dynamics
- Allosteric restriction elevates Fgr ATP K_m_ and boosts inhibitor potency
- Fgr conformational dynamics govern AML cell growth and bone marrow engraftment
