# Supplementary material for: Constraining regulatory domain dynamics of the Src kinase Fgr increases ATP-site inhibitor sensitivity and impairs bone marrow engraftment
Source: Cell Rep. Author manuscript; Available in PMC 2026 Jul 20. (PMC13383355; doi:10.1016/j.celrep.2026.117551)
Supplement: 1 [file NIHMS2190774-supplement-1.pdf]

Cell Reports, Volume *45*

Supplemental information

Constraining regulatory domain dynamics of the Src kinase Fgr increases ATP-site inhibitor sensitivity and impairs bone marrow engraftment

Giancarlo Gonzalez-Areizaga, Sherry T. Shu, John J. Alvarado, Haibin Shi, Li Chen, and Thomas E. Smithgall

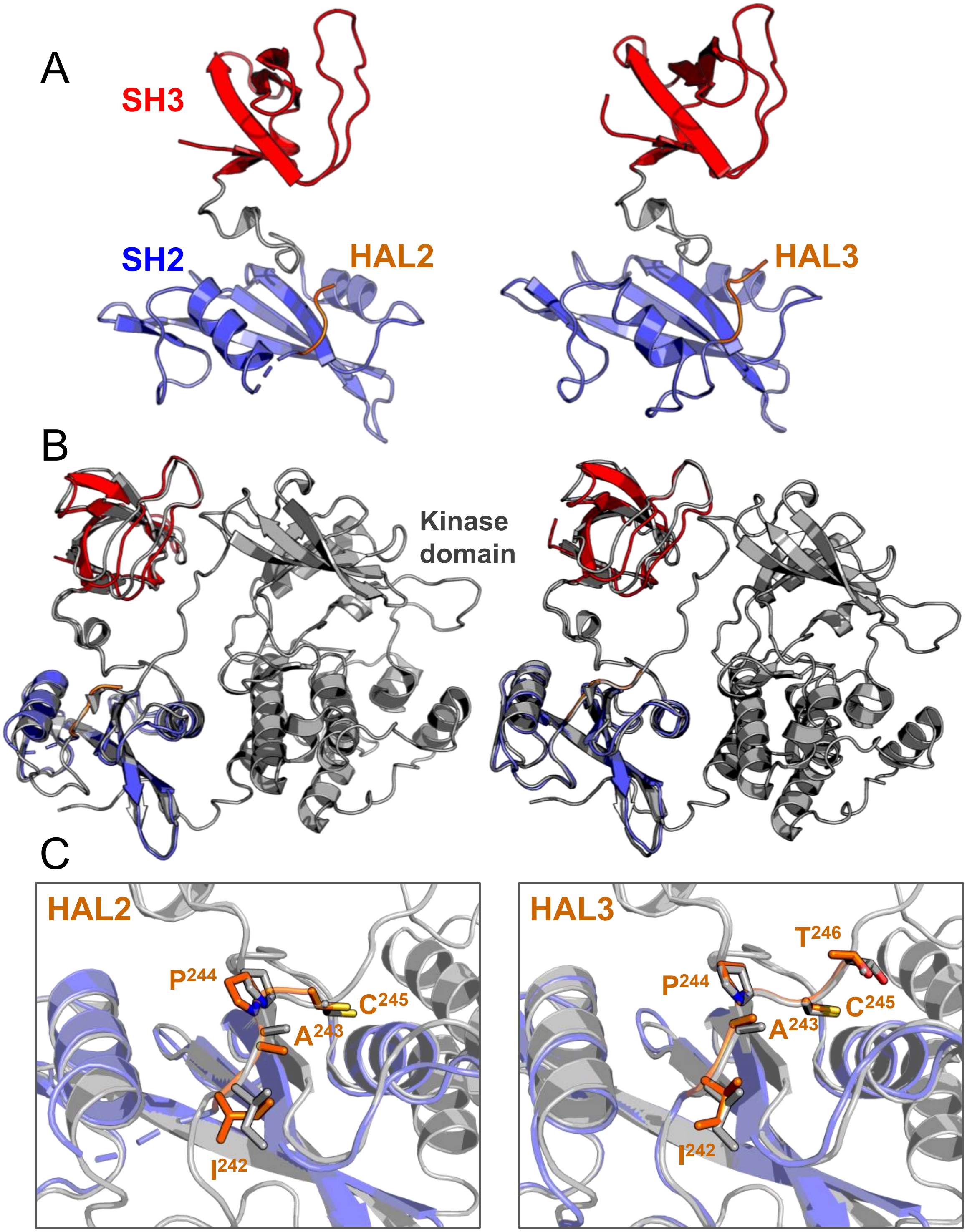

Figure S1. X-ray crystal structures of Fgr SH3-SH2-HAL2 and SH3-SH2-HAL3. A) Structures of Fgr SH3-SH2-linker (32L) proteins with high affinity linker variants 2 (HAL2, left) and 3 (HAL3; right). Shown are the SH3 domains (red), the SH3-SH2 connectors (grey), the SH2 domains (blue), and the N-terminal ends of the linkers (orange). Complete linker sequences were not resolved in these structures due to crystal contacts between Asp99 in the SH3 domain RT loop and Arg190 in the SH2 domain of a symmetry related molecule. B) Alignment of the Fgr 32-HAL2 (left) and 32-HAL3 (right) with the crystal structure of near-full-length Fgr in the closed conformation (PDB: 7UY0; rendered in grey). The SH3 and SH2 domains in both HAL structures as well as the partial linker sequence are oriented in a conformation compatible with the closed conformation of the overall kinase. HAL2 aligns with an RMSD of 1.08 Å over 148 atoms while HAL3 aligns with an RMSD of 0.83 Å over 132 atoms. C) Close in views of the HAL2 and HAL3 linker side chain (orange) alignments with the corresponding residues in near-full-length Fgr (grey). Despite the lack of the kinase domain, these linker residues adopt conformations like those in the closed conformation of Fgr, especially for HAL3.

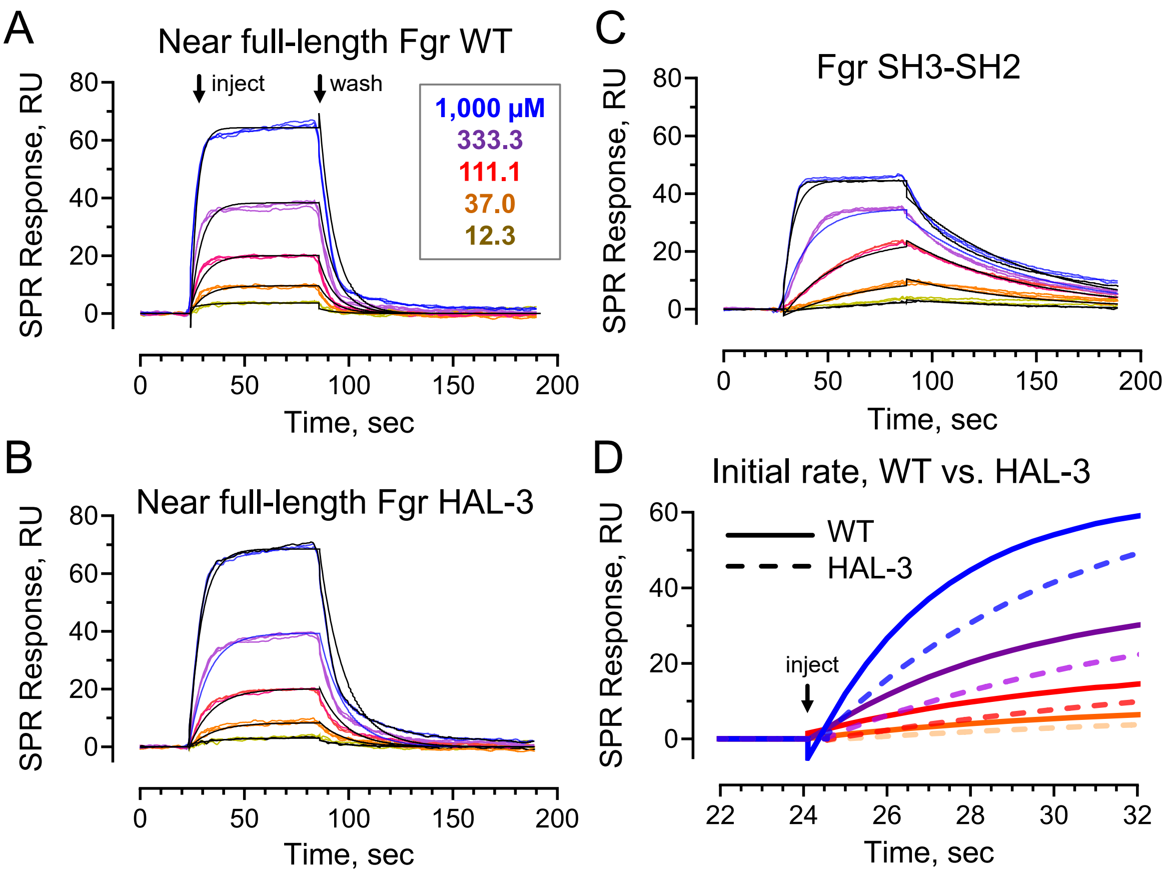

Protein	k_{on}, $M^{-1}s^{-1}$		k_{off}, s^{-1}		K_D, M		Chi^2	
	Run 1	Run 2	Run 1	Run 2	Run 1	Run 2	Run 1	Run 2
**WT**	1,960	3,090	0.131	0.132	6.7×10^{-5}	4.3×10^{-5}	1.35	1.47
**HAL-3**	844	1,060	0.082	0.076	9.8×10^{-5}	7.2×10^{-5}	1.80	2.16
**SH3-SH2**	1,840	2,370	0.017	0.017	9.5×10^{-6}	7.1×10^{-6}	1.09	0.87

Figure S2. High affinity linker (HAL) mutations enhance SH3:linker interaction in the context of near full-length Fgr. VSL12 peptide binding was assessed by surface plasmon resonance (SPR) to the SH3 domains of near full-length Fgr wild-type (WT, panel **A**) vs. HAL-3 (**B**) with Fgr SH3-SH2 as a positive control (**C**). Recombinant near-full-length Fgr proteins were produced in *E. coli* and consist of the SH3, SH2, and kinase domains and the phosphorylated C-terminal tail. Each Fgr protein was immobilized on the SPR chip and VSL12 was then injected in triplicate over the range of concentrations shown until steady-state was reached, followed by a dissociation phase (arrows). Representative sensorgrams are shown with the data in color and the fitted curves in black. The resulting kinetic constants from two independent experiments are shown in the table below the figure. In panel **D**, the fitted rate data from the initial association phases of WT and HAL-3 are overlaid to illustrate the slower on-rate of VSL-12 vs. HAL-3.

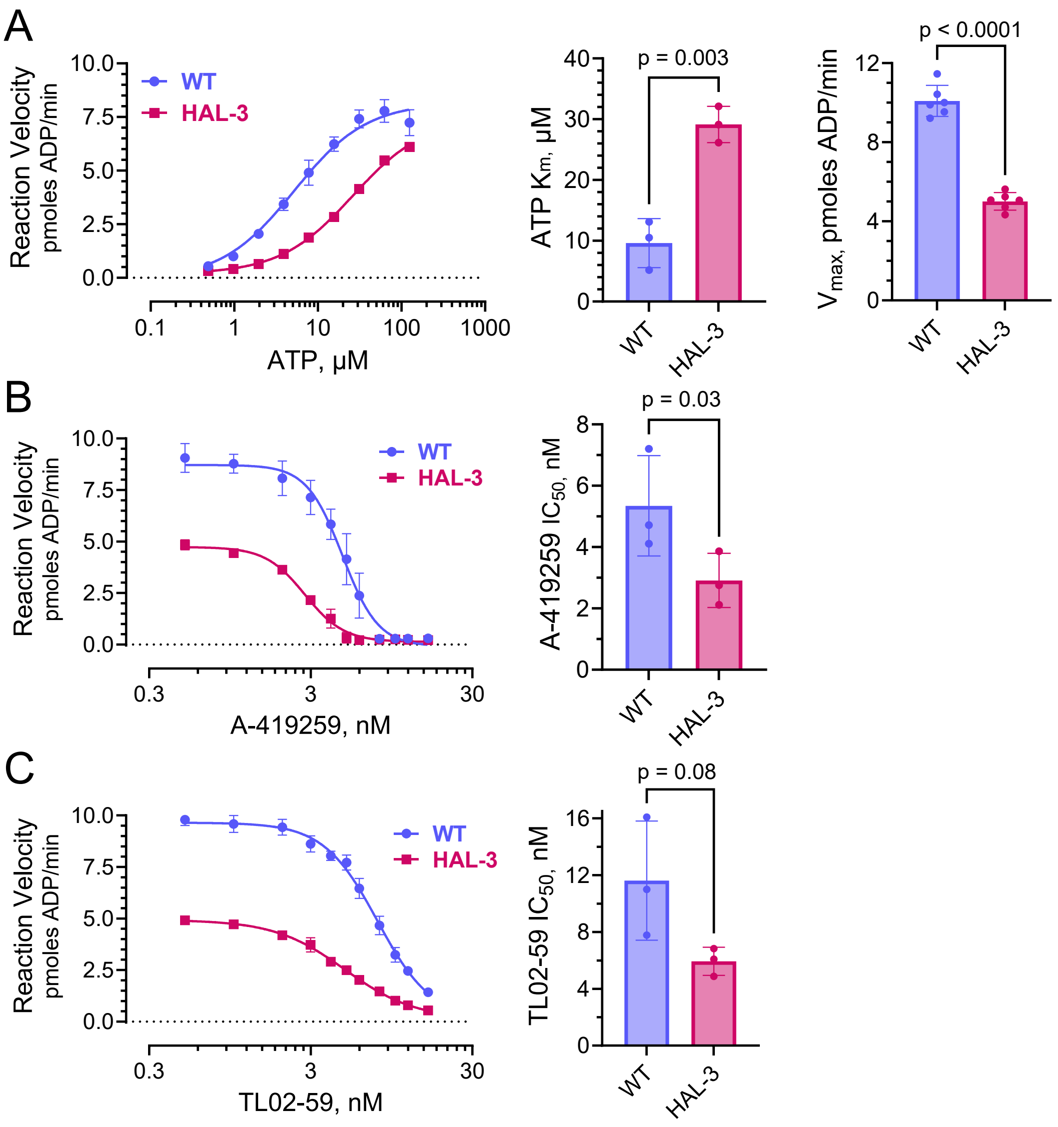

Figure S3. Introduction of HAL-3 in near full-length Fgr increases the K_m for ATP and enhances ATP-site inhibitor potency. Recombinant near-full-length Fgr proteins (wild-type and HAL-3) were produced in bacteria and purified to homogeneity in their tail-phosphorylated forms. Kinase activity was measured using the ADP Quest assay, which reports the generation of ADP from ATP as a function of time. **A**. Representative concentration-response curves (*left*) of ADP production as a function of ATP concentration at a constant protein kinase input of 58 nM per well per replicate. The apparent K_m and maximum enzyme velocity (V_{max}) values were determined from at least three independent replicates by non-linear curve fitting. The mean values are shown in the bar graphs (*center* and *right*) with individual values shown as points. Representative concentration-response curves for ADP production in the presence of ATP-site inhibitors A-419259 (**B**) and TL02-59 (**C**) at constant kinase and ATP concentrations (58 nM and 100 μM, respectively; *left*). IC_{50} values were determined from three independent replicates by non-linear regression. Mean values are shown in the bar graphs (*right*) along with individual points from three biological replicates. All bar heights indicate the mean ± SEM. Significance was evaluated by Student's t test and P values are shown above the bargraphs.

Table S1. X-ray data collection and refinement statistics for Fgr SH3-SH2-linker proteins with wild-type and high-affinity linkers (HALs). Statistics for the highest resolution shell are shown in parentheses.

	**Wild-Type**	**HAL-1**	**HAL2**	**HAL3**
**PDB ID**	10DT	10FS	10FV	10GA
**Data Collection**				
X-Ray source	APS 23-ID-B		APS 23-ID-D	
Resolution (Å)	38.55-1.80 (1.84-1.80)	39.12-1.30 (1.32-1.30)	42.65-1.80 (1.84-1.80)	60.08-1.60 (1.63-1.60)
Wavelength (Å)	1.03320			
Space group	$P2_1$			
**Cell dimension**				
a, b, c (Å)<br>α, β, γ (°)	44.185, 34.867, 60.109<br>90.00, 100.75, 90.00	44.443, 34.794, 61.677<br>90.00, 101.05, 90.00	43.393, 35.127, 60.846<br>90.00, 100.64, 90.00	44.613, 34.907, 61.315<br>90.00, 101.52, 90.00
Total reflections	74,879	606,680	32,715	303,258
Unique reflections	16,655	45,366	16,998	24,665
Mean (I/σ)	5.5 (1.7)	14.9 (0.90)	9.7 (1.1)	13.9 (2.3)
Redundancy	4.5 (4.3)	13.4 (13.2)	1.9 (2.0)	12.3 (8.6)
Completeness (%)	98.6 (97.4)	99.0 (98.8)	100.0 (100.0)	99.7 (95.9)
R_{merge}	0.231 (1.430)	0.068 (3.591)	0.029 (0.621)	0.130 (2.126)
CC(1/2)	0.981 (0.429)	1.000 (0.600)	0.999 (0.485)	0.999 (0.700)
**Refinement**				
Resolution (Å)	38.61-1.80	39.15-1.30	42.65-1.80	60.08-1.60
No. of reflections	15,839	43,020	16,104	23,435
R_{work}/R_{free}	0.166/0.207	0.181/0.199	0.194/ 0.233	0.169/ 0.194
**Number of non-H atoms**				
Protein	1,427	1,395	1,251	1,339
Solvent	190	174	111	134
**RMS deviations**				
Bonds Length (Å)	0.015	0.011	0.008	0.012
Bond Angles (°)	2.270	1.869	1.629	1.966
**Ramachandran**				
Favored (%)	98.00	97.75	99.00	98.00
Allowed (%)	2.00	1.69	1.00	2.00
Outlier (%)	0.00	0.56	0.00	0.00
**Average B factor (Å^2)**				
Protein	18.15	28.06	45.79	21.68
Solvent	42.60	35.60	45.70	35.10
